# Concentration Fluctuations from Localized Atmospheric Releases

**DOI:** 10.1007/s10546-020-00547-4

**Published:** 2020-08-04

**Authors:** Massimo Cassiani, Matteo B. Bertagni, Massimo Marro, Pietro Salizzoni

**Affiliations:** 1 NILU - Norwegian Institue for Air Research, 2027 Kjeller, Norway; 2 Department of Land, Infrastructure and Environmental Engineering, Politecnico di Torino, Corso Duca degli Abruzzi 24, 10124 Turin, Italy; 3 Laboratoire de Mécanique des Fluides et d’Acoustique, University of Lyon, CNRS UMR 5509 Ecole Centrale de Lyon, INSA Lyon, Université Claude Bernard, 36, avenue Guy de Collongue, 69134 Ecully, France

**Keywords:** Accidental hazards, Concentration fluctuation variance, Probability density function, Plume turbulent dispersion, Turbulent mixing

## Abstract

We review the efforts made by the scientific community in more than seventy years to elucidate the behaviour of concentration fluctuations arising from localized atmospheric releases of dynamically passive and non-reactive scalars. Concentration fluctuations are relevant in many fields including the evaluation of toxicity, flammability, and odour nuisance. Characterizing concentration fluctuations requires not just the mean concentration but also at least the variance of the concentration in the location of interest. However, for most purposes the characterization of the concentration fluctuations requires knowledge of the concentration probability density function (PDF) in the point of interest and even the time evolution of the concentration. We firstly review the experimental works made both in the field and in the laboratory, and cover both point sources and line sources. Regarding modelling approaches, we cover analytical, semi-analytical, and numerical methods. For clarity of presentation we subdivide the models in two groups, models linked to a transport equation, which usually require a numerical resolution, and models mainly based on phenomenological aspects of dispersion, often providing analytical or semi-analytical relations. The former group includes: large-eddy simulations, Reynolds-averaged Navier–Stokes methods, two-particle Lagrangian stochastic models, PDF transport equation methods, and heuristic Lagrangian single-particle methods. The latter group includes: fluctuating plume models, semi-empirical models for the concentration moments, analytical models for the concentration PDF, and concentration time-series models. We close the review with a brief discussion highlighting possible useful additions to experiments and improvements to models.

## Introduction and Motivation

Hazards and risks related to the atmospheric dispersion of pollutants continue to draw increasing attention within social, economic, and political issues. Over the years, the growing interest on this matter has been fed by the occurrence of major technological accidents (e.g., Seveso, Chernobyl, Bhopal, Fukushima), the increasing scientific evidence of the effects on human health of the exposure to indoor and outdoor air pollution (Loomis et al. 2013), and the risk of terrorist acts producing harmful releases in industrial sites, and in (indoor and outdoor) crowded public spaces. These concerns are today emphasised by the enhanced urbanization worldwide and the higher population density surrounding industrial districts. The proximity of industrial and residential sites represents a major concern not only for the population, but also for public authorities and industrial operators, whose business and activities may be adversely affected by strict regulations.

The atmospheric dispersion of pollutant is a phenomenon to which all of us are familiar, due to the ubiquitous presence in our everyday life of ‘smoke’ plumes emitted from industrial stacks, chimneys, car exhausts, biomass burning, or cigarettes. Without any need of specific scientific knowledge, the turbulent nature of these atmospheric releases is evident at first glance. A more attentive observation of the plume morphology can further reveal that its fluctuations are characterized by a wide range of temporal and spatial scales.

**Fig. 1:**
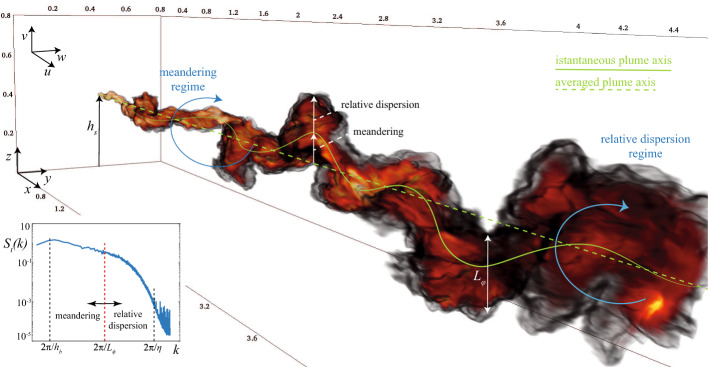
Volume rendering of turbulent dispersion of a passive scalar released from a point source as simulated in a large-eddy simulation ($2048 \times 512 \times 512$ nodes for a domain of $6h_b \times h_b \times h_b$, respectively in the along-wind, crosswind, and vertical directions). The blue arcs highlight two turbulent eddies of the same size. When the turbulent eddy is larger (smaller) than the plume size $L_\phi$, the eddy mostly contributes to meandering (relative dispersion). This is further highlighted in the turbulent energy spectrum $S_t(k)$ (left-low panel); $h_b$ and $\eta$ are the boundary-layer height and the Kolmogorov microscale, respectively

Indeed, a plume (or puff) of pollutant of generic size $L_\phi$ (see Fig. 1) released in a turbulent atmospheric flow is submitted to the action of eddies that can be larger than similar to or smaller than the plume size. These eddies will be efficient in very different ways in transporting the contaminant plume across the flow and mixing it with the ambient air. Following a well-established approach (Gifford 1959), this multiscale dispersion process can be described as the resulting action of two bulk phenomena: (i) the irregular motion of the centre of mass of the polluted fluid volumes, and (ii) a diffusive process due to the action of smaller scale eddies that acts in deforming and expanding the blob of marked fluid volumes and locally enhancing concentration gradients. The first process is referred to as meandering, whereas the second is referred to as relative dispersion, i.e., relative to the local centre of mass (Csanady 1973; Monin and Yaglom 1975). The relative importance of these processes depends on a large number of factors, namely the source size, the distance from the source of the observation point, the conditions imposed at the source, the thermal stratification of the atmosphere, and the geometry of the domain.

**Fig. 2:**
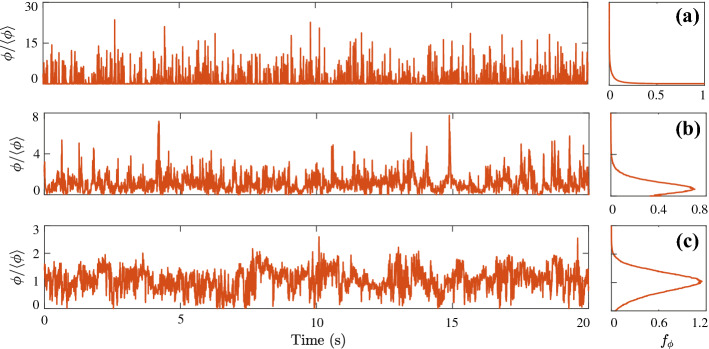
Concentration time series and related PDFs from the wind-tunnel experiments of Nironi et al. (2015). At increasing distance from the source, the PDF shifts from an exponential-like in the near-field (**a**), to a right-skew Gaussian-like PDF in the far-field (**c**)

These complex multi-scale dispersion mechanisms are finally reflected in the fluctuating character of a concentration signal recorded downstream of a pollutant source (Fig. 2). Based on the estimates of the statistical characteristics of these signals, we can effectively determine the impact of pollutant releases on health and the environment (and their related risks). Nevertheless, the level of accuracy of the statistical characterization depends on the typology of hazards that have to be assessed. In several problems, it is sufficient to estimate a time-averaged mean concentration ${\bar{\phi }}$ over a certain period of time ${\Delta }t$,

1 $$\begin{aligned} {\bar{\phi }} = \frac{1}{{\Delta } t} \int _{0}^{{\Delta } t} \phi (t) dt. \end{aligned}$$

This is typically the case for persistent hazards and risks associated with exposure to nitrogen oxides or particulate matter in air, since the accumulation process filters the effect of concentration fluctuations. A typical averaging time ${\Delta } t$ will range from a minimum of an hour, when considering acute respiratory and cardiovascular damages (Wong et al. 2008; Bhaskaran et al. 2011), up to one year, when dealing with long latency pathologies (Andersen et al. 2017).

Conversely, for the assessment of accidental hazards due to toxic or explosive airborne pollutants (inflammability and toxicity), knowledge of the mean concentration (Eq. 1) must be coupled with the probability of exceeding a specific concentration threshold and the expected mean time above the threshold (e.g., Hilderman et al. 1999; Gant et al. 2011; Gant and Kelsey 2012; Gunatilaka et al. 2014). Similarly, the impact of odours depends on instantaneous peak concentrations (e.g., Capelli et al. 2013; Sommer-Quabach et al. 2014), or concentrations averaged over the duration of one human breath (Mainland and Sobel 2006), to which the human nose is sensitive.

Quantifying the above-mentioned risks therefore requires the adoption of mathematical models to compute one-point concentration statistics. In principle, we could fully rely on the advection–diffusion equation

2 $$\begin{aligned} \frac{\partial \phi }{\partial t} + u_i \frac{\partial \phi }{\partial x_i} = D \frac{\partial ^2 \phi }{\partial x_i^2}, \end{aligned}$$

which exhaustively characterizes the evolution of the spatial and temporal distributions of the scalar concentration $\phi (\varvec{x},t)$ due to the action of the instantaneous turbulent velocity field $u_i$ and molecular diffusion *D*. Ideally, the Navier–Stokes equation for the velocity field and Eq. 2 can be solved by means of direct numerical simulation (DNS). Practically, this option is unfeasible because of the high Reynolds number $Re=U L /\nu$ in the atmosphere (Pope 2000), where *U* and *L* are characteristic velocity and length scales, respectively, and $\nu$ is the kinematic viscosity, which is here assumed equal to *D*, i.e. unitary Schmidt number $Sc=\nu / D\approx 1$. Indeed, the turbulent nature of the flow produces fluctuating velocity and concentration fields over a range of scales that cannot be covered by DNS. This can only be used as a guidance to understanding the underlying physical processes at relatively low *Re* (Vrieling and Nieuwstadt 2003; Rossi et al. 2010; Branford et al. 2011; Oskouie et al. 2017). The velocity and scalar concentration must then be treated as random fields (Monin and Yaglom 1975), by adopting some sort of averaging operator, i.e. time averaging ${\bar{\cdot }}$, ensemble averaging $\left\langle \cdot \right\rangle$, or volume averaging ${\tilde{\cdot }}$.

Adopting an averaging approach in order to filter out the smaller scale fluctuations (of both velocity and concentration fields) leads to the formulation of the so-called large-eddy simulation (LES) models. Contrary to all other methods available and discussed in this review, LES explicitly solves the three-dimensional variability of most of the turbulent structures (i.e., those larger than the filter size). In the case of LES (reviewed below in Sect. 3.1), the modelling of velocity and scalar field cannot be treated separately and the computational requirements are extremely demanding.

By applying an ensemble averaging operator to the advection–diffusion equation, Eq. 2, and using the Reynolds-averaging rule (leading to the Reynolds-averaged Navier–Stokes (RANS) method, e.g. Monin and Yaglom 1971; Tennekes and Lumley 1972; Stull 1988) we obtain a hierarchy of unclosed equations for the evolution of the concentration moments. The equation for the first moment, i.e., the mean, reads

3 $$\begin{aligned} \frac{\partial \left\langle \phi \right\rangle }{\partial t} + \left\langle u_i \right\rangle \frac{\partial \left\langle \phi \right\rangle }{\partial x_i} = -\frac{\partial \left\langle u_i' \phi ' \right\rangle }{\partial x_i} + D \frac{\partial ^2 \left\langle \phi \right\rangle }{\partial x_i^2}, \end{aligned}$$

where, as is customary, a prime represents a fluctuation from the mean value, i.e. $\phi '= \phi - \left\langle \phi \right\rangle$. On the right-hand side (r.h.s.), we recognize the well known problem of closure of the turbulent fluxes $\left\langle u_i' \phi ' \right\rangle$. Similarly, we can obtain the transport equation for the concentration variance $\left\langle \phi '^2 \right\rangle$ (hereafter also denoted $\sigma _{\phi }^2$), as presented in the seminal work of Csanady (1967),

4 $$\begin{aligned} \frac{\partial \left\langle \phi '^2 \right\rangle }{\partial t} + \left\langle u_i \right\rangle \frac{\partial \left\langle \phi '^2 \right\rangle }{\partial x_i} = -2 \left\langle u'_i \phi ' \right\rangle \frac{\partial \left\langle \phi \right\rangle }{\partial x_i} - \frac{\partial \left\langle u'_i \phi '^2 \right\rangle }{\partial x_i}-\varepsilon _{\phi }. \end{aligned}$$

The first term on the r.h.s. is the variance production, the second is the variance turbulent transport, and the third $\varepsilon _{\phi } = 2 D \left\langle \partial \phi '/\partial x_i \, \partial \phi '/\partial x_i \right\rangle$ is the variance dissipation, which controls the decay of concentration fluctuations. This last term is unclosed. According to the phenomenological description of the dispersion process outlined above, meandering is mainly linked to the production of scalar variance close to the source (e.g., Fackrell and Robins 1982b; Ardeshiri et al. 2020), while relative dispersion is mostly linked to its dissipation (e.g., Sykes et al. 1984; Cassiani et al. 2005a). Equation 4, its applications, and closures are further discussed in Sect. 3.2.

A more comprehensive description of the one-point concentration fluctuation statistics can be instead captured from the evolution equation for the concentration PDF $f_\phi$. This equation can be derived from the Navier–Stokes and the advection–diffusion equations and reads (e.g. Pope 1985, 2000)

5 $$\begin{aligned} \frac{\partial f_\phi }{\partial t} + \left\langle u_i \right\rangle \frac{\partial f_\phi }{\partial x_i}= - \frac{\partial }{\partial x_i} \left( f_{\phi } \left\langle u_i' \Big | \phi =\psi \right\rangle \right) -\frac{\partial }{\partial \psi } \left( f_{\phi } \left\langle D \frac{\partial ^2 \phi }{\partial x_i^2} \Big | \phi =\psi \right\rangle \right) , \end{aligned}$$

where $\psi$ is the sample space variable of the the random scalar field $\phi$ and $\left\langle \Big | \right\rangle$ denotes a conditional average. The first term on the r.h.s. is the flux of probability due the fluctuating velocity field. This is unclosed since $f_{\phi }$ does not include any information about the velocity, and is the equivalent of the unclosed turbulent flux in Eqs. 3 and 4 above. The second term on the r.h.s. is the conditional Laplacian which in high *Re* can be shown to be equivalent to the conditional scalar dissipation (e.g. Pope 2000, p. 546). This term defines the dissipation of scalar fluctuations and it is unclosed because $f_{\phi }$ does not contain any information about instantaneous spatial gradients. All these unclosed terms can be modelled, and the closure and solution of this equation is the subject of Sect. 3.3. From Eq. 5, the hierarchy of the unclosed RANS equations for the evolution of the concentration moments can be obtained by integration (e.g. Pope 2000, p. 553).

Mathematically, the last terms in Eqs. 4 and 5 represent a sink of scalar variance and fluctuations, respectively. Physically, these terms model the mixing of the scalar with the ambient air, which is the result of the complex motion of blobs of polluted fluid within the ambient fluid. Strictly speaking, the *mixing* is a phenomenon that takes place at the molecular scale, and is therefore driven by local instantaneous concentration gradients. However, in very high *Re* flow, the intensity of the gradients (and therefore of mixing) is governed by the multi-scale dynamics of the turbulent flow, which folds and stirs the scalar field until gradients are so large that molecular mixing becomes effective, and not by the molecular diffusivity itself. This leads to the apparent paradox that the modelling of scalar dissipation does not explicitly involve molecular diffusivity. This feature is further discussed in Sect. 3.4, where the use of two-particle Lagrangian stochastic models for concentration fluctuations is reviewed. Note that the link between mixing and two-particle models shows that the main time and length scales characterizing the scalar dissipation are those of the relative dispersion process (e.g., Sykes et al. 1984; Thomson 1990, 1996; Sawford 2001; Cassiani et al. 2005a).

So far, we have introduced methods that make use of transport equations and minimal closure assumptions for the time and space variations of the concentration PDF or its lower order moments. Beside these, other approaches have been proposed in the literature, based on phenomenological aspects and experimental evidence. These have the main advantage of being computationally much more efficient. Gifford (1959) was the first author to use the phenomenological concepts of meandering and relative dispersion to propose an analytical model that, as far as we are aware, is the first quantitative model of concentration fluctuations. From his seminal work stemmed a number of models, referred to as *fluctuating plume models*, that are discussed in Sect. 4.1. Even simpler empirically-based models for the concentration moments were obtained starting from arguments about mixing or simplifications of the concentration variance equation (Eq. 4). These models, which have the advantage of being in analytical closed form, are discussed in Sect. 4.2. There is also a class of heuristic numerical models that make use of Lagrangian one-particle dispersion models and heuristic mixing relationships based either on the RANS (Eq. 4), or PDF (Eq. 5), transport equations to estimate the concentration variance. These are presented in Sect. 3.5.

Instead of solving a closed form of Eq. 5, the shape of $f_\phi$ can be reconstructed by means of analytical models, which require knowledge of only a few moments of concentration, usually the mean and the variance. These can be obtained through any of the empirical or numerical modelling methods introduced above. The formulation of analytical models for the PDF shape is reviewed in Sect. 4.3 and strongly relies on the comparison with experimental measurements.

Finally, we mention the *time-series* models (Sect. 4.4), devoted to the evaluation of threshold upcrossing rates. They are conceptually different from all other methods discussed above, since they require information about the time dynamics of the concentration field, which are not included into the PDF. This information is usually reconstructed through the use of a limited number of spatial and temporal characteristic scales.

The development of all above-mentioned modelling approaches (from the adoption of specific closure relations for Eqs. 4 and 5 to the formulation of simplified heuristic models) relies on our understanding of the physical mechanisms that govern the turbulent mixing of scalars emitted from localized sources. Historically, understanding these mechanisms has heavily relied on experiments, performed both in the field and in the laboratory. These are extensively reviewed in Sect. 2. The experimental results also provide the essential data needed to test the reliability of each model.

To date, due to its scientific and operational relevance, turbulent scalar dispersion has been the subject of several reviews (e.g. Hanna 1984b; Weil 1995; Wilson 1995; Warhaft 2000). The significant scientific advances in the last 20 years motivate in our opinion a novel and updated review, in which we specifically consider non-buoyant scalar releases from localized sources. For a broader view on the physical processes underlying turbulent mixing (without any focus on the effects induced by a localized release) the reader is referred to the reviews by Shraiman and Siggia (2000), Dimotakis (2005), Sreenivasan (2018), and Villermaux (2019).

**Table 1:** Resume of the different approaches to evaluate the concentration fluctuation statistics

Approach	Sect.	Statistics				Known applications		
		$\mu _2$	$\mu _{3,4}$	PDF	$N^+_\theta$	Neutral	Conv.	C. geom.
LES	3.1	O	O	O	O	x	x	x
RANS	3.2	O				x		x
PDF methods	3.3	O	O	O		x	x	x
2-Particle	3.4	O				x		
Lagrangian heuristic	3.5	O				x	x	x
Fluctuating plume	4.1	O	O	I/O		x	x	
Empirical relations	4.2	O	O			x		
Analytical PDF	4.3	I	I/O	O		x		
Time series	4.4	I	I/O	I	O	x		

The applicability of every approach is also mentioned: neutral and convective (Conv.) boundary layers, and Complex Geometry (C. geom.). We discriminate between output (O) and input (I) of the approach; $\mu _i$ is the *i*-statistical moment of concentration and $N^+_\theta$ is the upcrossing rate (the mean frequency of exceeding a concentration threshold $\theta$, see Sect. 4.4). In fluctuating plume models, the concentration PDF in relative coordinate is an input, but the overall concentration PDF can be an output, see Sect. 4.1

The purpose of this introduction is to provide a brief overview on the variety of modelling methods that are today available to estimate concentration fluctuations, see also Table 1. In what follows, we first deal with the field and laboratory experiments (Sect. 2), and then group the modelling methods into two main classes: (i) models linked to a transport equation, which usually requires a numerical solution, and (ii) models mainly based on phenomenological aspects of dispersion, often providing analytical or semi-analytical relations. The former are presented in Sect. 3, and include: LES, RANS, two-particle Lagrangian stochastic models, PDF transport equation methods, and heuristic Lagrangian single-particle methods. The latter are presented in Sect. 4 and include: fluctuating plume models, semi-empirical models for the concentration moments, analytical models for the concentration PDF, and, finally, concentration time-series models. There may undoubtedly be overlap between these two categories. Nonetheless, we believe this classification to be the best discrimination among the different modelling approaches.

In the following, meteorological or index notation are used when convenient, so $u_1=u, u_2=v, u_3=w$ represent the velocity components in the along wind $x_1=x$, crosswind $x_2=y$, and vertical $x_3=z$, directions respectively. Vectors are represented in bold character, e.g. $\varvec{x}=(x_1,x_2,x_3)$, and Lagrangian quantities with a star, e.g., $\varvec{x}^*$.

## Experiments

In the 1950s and 1960s seminal experiments in open terrain produced a first insight into the framework of concentration fluctuations from a qualitative point of view. These experiments essentially considered dispersion in the atmospheric surface layer due to releases from point sources placed close to the ground (Sect. 2.1.1). Laboratory studies of passive scalar releases arose later in the literature, while early wind-tunnel experiments appeared essentially from the late 1970s. Differently from open field experiments, laboratory experiments allowed for the investigation of a wider typology of source condition, including point (Sect. 2.2.1) and line sources (Sect. 2.2.2) of variable size and position (with respect to the ground) and in different flow typologies (grid turbulence, channel flow, boundary layer). At the same time, laboratory experiments have been mainly limited to neutral flows and have been rarely performed for non-neutrally stratified boundary layers (Sect. 2.2.3). The interest of the scientific community shifted subsequently, from around 2000, to the dispersion within urban areas that have been investigated by means of both field (Sect. 2.1.2) and laboratory experiments (Sect. 2.2.4).

### Field Experiments

#### Open Field

Initially, field experiments have been mainly devoted to estimating the peak-to-mean concentration ratio (Lowry et al. 1951; Gosline 1952; Singer 1961), and investigating also the effects of complex terrain and vegetation (Singer et al. 1963), and of buildings (Hinds 1969).

Subsequent studies were devoted to a deeper characterization of the scalar field and provided estimates of the higher-order statistics. Notably experiments by Ramsdell and Hinos (1971), Hanna (1984a), and Dinar et al. (1988) provided estimates of fluctuation intensity ($i_\phi = \sigma _{\phi } / \left\langle \phi \right\rangle$), skewness, kurtosis, the intermittency factor (fraction of time for which $\phi >0$), and PDF, and Hanna and Insley (1989) presented concentration spectra. Mylne and Mason (1991) and Mylne (1992) investigated concentration fluctuations from ground-level or slightly elevated sources, for a wide range of downwind distances (45 to 1000 m). Experiments by Mylne and Mason (1991) were performed in near-neutral to convective conditions, whereas those by Mylne (1992) in stable conditions. In both cases, however, the authors could not identify any specific effect of atmospheric stability on concentration statistics. Mole and Jones (1994) also investigated the role of atmospheric stability, both stable and unstable, performing measurements over a much shorter range of downwind distances than those considered by Mylne and Mason (1991) and Mylne (1992). The influence of stability conditions was detected in estimates of skewness and kurtosis but not so significant as to modify the shape of the PDF. Mylne (1993) extended his previous analysis to vertical profiles of concentration fluctuations measured at two downwind distances (50 m and 100 m). He presented an exhaustive statistical analysis of concentration statistics, including estimates of characteristic scalar length scales. Interestingly, he noted that, at the considered distances, sources at or near the ground behaved differently from those at 4 m height. By comparing the concentration fluctuation statistics obtained from fast and relatively slow sensors Mylne et al. (1996) showed the importance of high frequency measurements in characterizing concentration peaks close to the source.

A significant contribution was given by the experimental works performed by Yee and co-workers in the framework of the CONFLUX (concentration fluctuation experiments) project, which involved three defence research establishments in the USA, UK, and Canada (Yee et al. 1993b, 1994c, d, 1995). The experimental campaigns focused on the measurements of time series of concentration by means of a high-frequency photoionization detector. The gas tracer was propylene ($\hbox {C}_3 \hbox {H}_6$), released from a point source for stable, near-neutral, and unstable stability conditions. They investigated the spatial evolution of the scalar statistics, i.e., fluctuation intensity, skewness, kurtosis, intermittency factor, and one-point PDF. More specifically, Yee et al. (1993b) tested five models (exponential, clipped-normal, lognormal, gamma, and Weibull) for the one-point concentration PDF and found that the lognormal and gamma distributions gave the best agreement, respectively, at short and far ranges. Yee et al. (1994d) investigated the same source configuration analyzing the spatial evolution of the concentration PDF. They observed that close to the source the PDF assumed an exponential-like shape, in the intermediate field it became bimodal, and in the far-field it had a unimodal structure. They argued that this behaviour reflected the interaction between the meandering and the relative dispersion. Finally, Yee et al. (1995) completed the analysis of the fluctuating plume by providing estimates of the mean dissipation rate of concentration variance as well as time and length scales related to the dissipation process. Other work focused on statistical investigations of the scalar field induced by the emission of quasi-instantaneous clouds using ensemble averaging. Yee et al. (1994c) showed that PDFs of maximum instantaneous concentration and dosage were modelled by a gamma distribution, whereas Yee et al. (1998) observed that the temporal autocorrelation of the concentration fluctuations was well approximated by a self-similar exponential function.

All the field measurements mentioned above used point-wise measurement systems sampling at high frequency. A different approach was taken by Lewellen and Sykes (1986a) who used a lidar system but with a quite coarse sampling volume and rate. More recently, in a series of field campaigns performed under the COFIN (concentration fluctuations in gas releases by industrial accidents) project by the Risø National Laboratory and Sheffield University, a lidar system was used (Jørgensen and Mikkelsen 1993; Mikkelsen et al. 2002) with a relatively low sampling frequency (0.33 Hz) but taking instantaneous crosswind sections with sampling length of about 1.4 m. This method allowed also measuring directly the concentration fluctuations in the coordinate system relative to the plume centre of mass (Munro et al. 2003). All the crosswind sections were taken far from the source and the vertical position of the source ranged from near-ground level to a height of 21 m (Munro et al. 2003). The data collected in these campaigns were used in a series of works exploring the shape of the concentration PDF and the modelling of extreme values (see Sect. 4.3 below). An alternative remote sensing approach based on multiple cameras was also implemented very recently with the aim of investigating concentration fluctuations, but so far results are limited to relative dispersion statistics (Dinger et al. 2018).

In the Project Sagebrush, continuous releases of $\hbox {SF}_6$ as a gas tracer were used in stable and unstable conditions at very low wind speed (Finn et al. 2018). The aim was to replicate and extend the results of the Prairie Grass experiment (Barad 1958), which was limited to the analysis of time-averaged concentrations. Finn et al. (2018) analyzed short-range plume dispersion using a large number of detectors (1 Hz sampling frequency) for evaluating both velocity field and scalar concentrations. The study focused on the concentration variability during the sampling operations, and the analysis of the time series showed that the meandering was a main source of uncertainty.

Odour plumes were specifically treated by Barynin and Wilson (1972), who compared the sensitivity of a fast-response flame-photometric detector with that of the human nose in detecting concentration fluctuations. Always dealing with odour plumes, Murlis et al. (2000) related the response of some insects, such as moths, to the concentration fluctuations of pheromones. He also compared the response of the antennae of a gypsy moth (electronantennograms) to the concentration time series collected through ion detectors in open terrain and forests. For a broader view on applications of the fluctuating plume theory for entomological studies, the reader is referred to, e.g., Murlis et al. (1992).

Finally, we mention the experiments concerning the interaction of multiple sources on the concentration statistics. Sawford et al. (1985) studied concentration fluctuations induced by isolated and multiple sources in unstable and neutral atmospheric boundary layers. They used two different gas tracers, $\text{ SF}_6$ and phosphorous, released from different sources in order to evaluate the concentration contributions at a given point from each of the sources. These measurements allowed the authors to compare and model the joint statistics in terms of those provided by the single sources. Davies et al. (2000) performed high-frequency concentration measurements in Cardington, UK, and in Nevada, USA. They used an ultraviolet ion collector and a flame ionization detector in order to measure the concentration fluctuations of ammonia and propane plumes, respectively, and analyzed the behaviour of the fluctuation intensity, the correlation due to the interaction of the two sources, and the PDF shape.

#### Urban Areas

The first significant urban campaign was that in Salt Lake City, using ground-level point and line sources of $\hbox {SF}_6$ (Allwine et al. 2002). The dataset was subsequently used by Chang et al. (2005) to evaluate the performance of a dispersion model with the scope of estimating maximal concentration values for hazard assessments.

In the Joint Urban 2003 experiment (JU2003), steady plumes and instantaneous puffs of $\hbox {SF}_6$ were released in Oklahoma City (Clawson et al. 2005) and measured using fast-response analyzers in both daytime and night-time conditions. A large variety of concentration statistics, including fluctuation intensities, peak-to-mean ratio, concentration percentiles, and intermittency factors were more recently discussed by Klein and Young (2011). They also showed that the two-parametric gamma and three-parametric clipped-gamma cumulative probability presented good agreement with the observations, particularly in the upper tail of the distributions. Further analysis of the JU2003 dataset was presented by Finn et al. (2010), who showed that the higher-order statistics of the concentration were more significant in daytime than in night-time. Finn et al. (2010) also verified that the lognormal distribution performs better than the exponential or the clipped-normal distributions in simulating the concentration statistics.

Santos et al. (2005) described a field experiment in the vicinity of a complex-shaped building, presenting values of mean and standard deviation and intermittency factor of the concentration. They emphasized the role of stability conditions and street geometries in affecting the concentration statistics.

Finally, Biltoft (2001) and Yee and Biltoft (2004) described experiments within an idealized urban geometry made up by a regular array of obstacles. The experiments, widely known as the Mock Urban Setting Trial (MUST), provided a unique dataset of velocity and concentration statistics: fluctuation intensity, peak-to-standard deviation ratio, probability density function, spectra, as well as estimates of characteristic time and length scales of dominant motions in the array plume (e.g., the integral scale, the Taylor microscale).

### Wind Tunnel and Flume Channel

#### Point Sources

An early experiment was that by Gad-el Hak and Morton (1979), who presented results from a point-source release in an isotropic turbulent flow. By combining laser Doppler velocimeter and a laser light-scattering technique, Gad-el Hak and Morton (1979) measured simultaneously one-point statistics of concentration and velocity, reporting the downstream evolution of the intermittency factor, the concentration fluctuation intensity, and the velocity–concentration correlations. Yee and Wilson (2000) discussed the results a saline plume dispersing in grid turbulence in a water channel and reported all the relevant velocity and concentration statistics, including the concentration PDF. They measured both vertical and crosswind profiles at several downwind distances. Brown and Bilger (1998) investigated the dispersion of a reactive plume of NO in a background of $\hbox {O}_3$ in decaying grid turbulence, and using conserved scalar theory they could also provide concentration moments up to the fourth order for the conserved scalar.

Almost all other experiments were instead performed in turbulent boundary layers, with both ground-level and elevated sources. One of the earliest work was the comprehensive monograph of Netterville (1979), who investigated a dispersing plume of helium using a hot-film concentration detector. The measurements were very comprehensive including velocity and scalar fluctuations, PDF, and the terms in the variance budget equation for both crosswind and vertical profiles at several downwind distances. Soon after, the highly cited experiments described in Fackrell and Robins (1982a, (1982b) were performed; these have been used over the years as reference test cases for the validation of a wide variety of numerical models. Beside the characterization of turbulent fluxes from elevated and ground-level sources, Fackrell and Robins discussed phenomenological and dynamical aspects of the dispersion and the effects of the source size and of the source elevation. As with Netterville (1979), they also evaluated the terms composing the concentration variance balance, including the variance dissipation. Their study was recently replicated by Nironi et al. (2015), who focused on higher-order concentration moments and deepened the analysis on the shape of the one-point concentration PDF, which was shown to be very well modelled by a gamma distribution.

A decade after these seminal works, Bara et al. (1992) investigated the structure and development of vertical and crosswind profiles of the mean, variance, intermittency, and conditional intensity of non-zero fluctuations using water-tank experiments. Bara et al. (1992) also interpreted their results through a plume fluctuating model. Liao and Cowen (2002) used a coupled PIV–LIF (particle image velocimetry–laser-induced fluorescence) system in a water channel to measure the velocity and scalar fields of a plume released by a point source within a turbulent boundary layer. In order to explain the ability of some animals, e.g., moths and crabs, in tracking a plume to its source, they analyzed the reliability of an inversion algorithm, using estimates of different concentration statistics as the reference variable. Xie et al. (2007) analyzed extreme concentration values of both elevated and ground-level point releases over rough boundary-layer flows. Their results for ground-level sources were shown to be in agreement with the field experiments of Mylne and Mason (1991), and suggested that the relative intensity of the fluctuations approaches a constant value in the far-field. In analyzing the occurrence of concentration peaks, Xie et al. (2007) highlighted the different roles of turbulence structure on elevated and ground-level sources.

Hilderman and Wilson (2007) performed experiments in a water channel using laser-induced fluorescence to characterize the meandering motion of a plume dispersing in a turbulent flow. According to their results, the centroid position of the crosswind concentration profile is characterized by a Gaussian probability density function, whereas the the fluctuation instantaneous plume spread about the centroid follows a lognormal distribution.

As far as we are aware, one single study has considered the dispersion from multiple point sources (Yee et al. 2003), who performed experiments in grid turbulence within a flume channel with two point sources. They quantified the spatial distribution of the one-point concentration PDF as well as the second-order correlation function.

#### Line Sources

Considering line sources, several experiments investigated the case of scalar dispersion in decaying grid turbulence (Warhaft and Lumley 1978; Warhaft 1984; Stapountzis et al. 1986; Sawford and Tivendale 1992). A first experiment was that of Warhaft and Lumley (1978), who investigated the decay of temperature fluctuations produced by a heated wire. Warhaft (1984) investigated the fluctuations due to a single and multiple (up to four) heated wires. He focused on the scalar variance in order to evaluate the second correlation functions. Stapountzis et al. (1986) measured the spatial distribution of concentration and one-point concentration PDF and spectra. A similar experiment was also performed by Sawford and Tivendale (1992), whose measurements were reported in detail in Sawford and Sullivan (1995) and included both along-wind and crosswind variations of concentration moments up to the fourth order.

Raupach and Legg (1983) investigated the dispersion from an elevated line source in a rough turbulent boundary layer, coupling a hot-wire anemometer and a cold-wire resistance thermometer. They presented an exhaustive picture of the mean and fluctuating concentration field, and reported centreline relative intensity for varying source diameter. Furthermore they focused on the scalar variance as well as the velocity–scalar correlation budgets. The same approach was extended to the case of line and plane sources within a modelled plant canopy, as discussed in Coppin et al. (1986) and Legg et al. (1986) who reported concentration statistics up to the fourth order.

Karnik and Tavoularis (1989) considered the case of a uniform sheared turbulent flow. They investigated the general structure of the fluctuating plume and focused on the re-appearance of variance peaks far downstream of the source as a result of variance fluxes induced by large-scale eddies.

Vinçont et al. (2000) investigated the dispersion of a line-source plume downwind of a surface-mounted two-dimensional cross-flow obstacle. Using optical techniques both in a water flume and a wind tunnel, they reported the spatial evolution of the standard deviation of concentration and focused on higher-order parametrization of velocity–concentration correlations. More recently, Lavertu and Mydlarski (2005) performed experiments in a developed channel flow with line sources and analyzed the shape of the concentration PDF and the distribution of scalar variance, depending on source size and position.

#### Non-neutral Flows

Dispersion in thermally-stratified flows has been rarely investigated in laboratory experiments. A significant step forward was made by the pioneering work of Deardorff and Willis (1984), who investigated concentration fluctuations within a convective boundary layer. The experimental set-up was a water tank heated from below, without any mean motion of the fluid in the horizontal direction. The effect of mean advection was then reproduced by a steady motion of the source within the tank, a method that of course does not allow for the simulation of the effects of the shear of the mean motion or of bottom friction. With this set-up they measured the decay of concentration fluctuation intensities along the plume centreline and showed the reliability of the gamma distribution for the concentration PDF. With a similar experimental set-up, Weil et al. (2002) investigated the dispersion of buoyant plumes in a convection tank. In contrast to Deardorff and Willis (1984), a laser was mounted on a movable table alongside the tank and towed at the stack speed in order to illuminate a crosswind-vertical plane at a fixed distance downstream of the stack. With this approach, they provided a set of measurements of all dispersion components, meandering, relative dispersion, and total dispersion, and discussed the (increased) centreline decay of concentration fluctuation intensity with respect to the neutral non-buoyant case, as reported by Fackrell and Robins (1982a). Finally, Marucci and Carpentieri (2020) recently investigated the effect of stable and convective atmospheric conditions on the mean and variance of the concentration within and above an obstacle array. As far as we are aware, no other experiments have so far investigated the concentration fluctuations within stably stratified flows.

#### Urban Mock-Up

As with field experiments, in recent years the focus of laboratory experiments has progressively shifted to the investigation of localized releases within groups of obstacles representing simplified urban mock-ups. A first work on urban-like geometries was that of Pavageau and Schatzmann (1999), who characterized the spatial distribution of concentration variance within an isolated two-dimensional street canyon.

A significant body of work on three-dimensional geometries was performed by Gailis and Hill (2006) and Gailis et al. (2007), who investigated the dispersion of a tracer within a large array of obstacles. They reproduced at the 1:50 scale the MUST experiment (Biltoft 2001; Yee and Biltoft 2004) in a water channel and used laser-induced fluorescence to measure the fluctuating concentration field. Gailis and Hill (2006) reported a wide range of concentration statistics and discussed similarities and differences between the full and small-scale experiments. Gailis et al. (2007) obtained the time series of plume centroid locations and the dispersion in the relative frame of reference. They showed that the PDF of the centroid horizontal position is well fitted by a Gaussian distribution, whereas the motion in the vertical direction is lognormally distributed. Furthermore, they found that the relative concentration PDF is well approximated by a gamma distribution. This dataset contained also the profiles of the fluctuation intensity in the relative coordinate in the *x*, *y*, and *z* directions. Other interesting small-scale reproductions of field experiments were presented by Arnold et al. (2004) and Klein et al. (2011). Arnold et al. (2004) provided an overview of the wind-tunnel experiments simulating the London site of the dispersion of air pollution and its penetration into the local environment (DAPPLE) project, which includes measurements of concentration fluctuations and turbulent fluxes (the latter were specifically analyzed by Carpentieri et al. 2012). Klein et al. (2011) simulated in a wind tunnel the Joint Urban 2003 experiments (Clawson et al. 2005) and showed the correspondence between the 98-percentile concentrations recorded in full-scale releases and those observed in the laboratory.

Other recent works on urban-like geometries include the already cited study of Marucci and Carpentieri (2020) and that of Di Bernardino et al. (2019) who, however, mainly focused on the determination of the turbulent Schmidt number rather than the concentration fluctuations.

## Transport Equation Methods

Studying the turbulent transport of pollutants in environmental flows requires a link with the statistics of the velocity field. Yet, as already mentioned above, LES is the only approach considered here in which the stochastic variability of the turbulent flow is explicitly solved. In all other approaches, the statistical description of the velocity field is assumed as input data for the problem. This description can be relatively simple when assuming that the velocity field can be represented as a boundary-layer flow over a rough surface. In that case, the (horizontally homogeneous) velocity statistics can be reconstructed through similarity relationships using local meteorological inputs (e.g. Stull 1988; Rodean 1996). The description of the velocity field is, however, much more difficult when dealing with flows in complex terrain and/or within heterogeneous urban (or industrial) geometries. Depending on the approach adopted to study the transport process, the information needed to statistically characterize the flow may be limited to the spatial distribution of the mean velocity $\left\langle u_i \right\rangle$, the turbulent kinetic energy $E=\left( 1/2\right) \left\langle u_i' u_i' \right\rangle$, its dissipation rate $\varepsilon$, or even the variances and cross-correlations, $\left\langle u_i' u_j' \right\rangle$, and third-order velocity moments. Estimating the relevant turbulent time and length scales is also usually needed.

The review of the different methods adopted for the diagnosis or prognosis of velocity fields is beyond the scope of our work. For this reason, we do not systematically go into details on the way that these key flow variables can be estimated, assuming that a method “exists” to obtain the required flow statistics.

### Large-Eddy Simulation

Large-eddy simulation explicitly simulates the most energetic part of the turbulent spectrum, while smaller scales, subfilter or subgrid, are parametrized using a subgrid-scale (SGS) model (e.g. Deardorff 1973; Moeng 1984; Pope 2000). In this way, LES gives access to the full three-dimensional and temporal variability of the (resolved) turbulent flow. However, this is at the expense of a formidable computational requirement as the velocity field needs to be simulated with a high resolution. Moreover, LES is necessarily unsteady and long time averaging is needed to obtain reliable statistics. In many real world applications, requiring large computational domains, LES is often used at the limit of the available computation resources. Issues therefore arise because the filter is defined to be equal or close to the grid size. Because of this, in solving the velocity field, LES results exhibit a dependence both on the grid resolution and on the numerical methods (e.g., Pope 2004; Geurts 2006; Kemenov et al. 2012). Avoiding these issues requires a clear gap between the filter width (and related mixing length in the SGS model) and the grid size (e.g., Mason and Callen 1986), a solution that is however rarely adopted. Further specific issues arise when simulating the scalar field, since this requires different numerical methods and SGS models (e.g. Colucci et al. 1998; Mironov et al. 2000; Balarac et al. 2008; Kaul et al. 2009; Heinze et al. 2015).

As shown in by laboratory experiments (e.g. Fackrell and Robins 1982a, b; Nironi et al. 2015) the ratio between the size of the source and that of the larger scale eddies has a great impact on the concentration statistics. This feature makes the LES results on concentration statistics particularly sensitive to the grid resolution (Ardeshiri et al. 2020). To avoid this, the size of the grid cell should be much smaller compared to that of the source, a condition that has not been adopted in most of the studies published so far.

Imposing this gap was clearly not affordable in the early studies, e.g., of Henn and Sykes (1992) and Sykes and Henn (1992b), due to the limited computational resources available at that time. In studying dispersion in the convective and neutral boundary layers, they simulated the effect of sources smaller than the grid size by using a SGS puff model. The puff expansion was based on a parametrization proposed by the same authors in previous RANS simulations (Sykes et al. 1984; Sykes and Henn 1992a). Results for the concentration variance, $\sigma _{\phi }^2$, and relative intensity of concentration fluctuations, $i_{\phi }$, were compared with the measurements of Deardorff and Willis (1984) and Fackrell and Robins (1982b) in convective and neutral conditions, respectively. The simulated cumulative distribution functions were also reported and qualitative comparisons with lognormal and clipped-normal distributions included.

More than a decade later, taking advantage of increased computational power, Xie et al. (2004, (2007) were able to simulate the same case study (plume dispersion in a neutral boundary layer) with a higher grid refinement and resolving the scalar source by one grid cell. In the scalar balance equation, they used the SMART (sharp and monotonic algorithm for realistic transport, Waterson and Deconinck 1995) method to discretize the advection term and adopted no SGS model for the small-scale fluctuations. Despite the grid resolution being still limited (compared to the source size), the results of Xie et al. (2004, (2007) for $\sigma _{\phi }$ and $i_{\phi }$, were in satisfactory agreement with their own experimental results (Xie et al. 2004, 2007) and those of Fackrell and Robins (1982b).

Around that time, Dosio et al. (2003) and Dosio and de Arellano (2006) investigated dispersion in the core of the planetary convective boundary layer. Adopting a periodic domain (both in streamwise and cross-wind directions), they simulated a steady point source by means of an instantaneous line source (and using the Taylor frozen turbulence hypothesis to transform the time after the initial release into the distance downwind the source). The source size measured two grid spacings vertically and one grid spacing horizontally in Dosio et al. (2003) and one grid spacing in Dosio and de Arellano (2006). No quantitative comparison with experimental results was attempted in Dosio et al. (2003), while Dosio and de Arellano (2006) showed instead a good agreement with the ground-level concentration fluctuations measured in Deardorff and Willis (1984) and Weil et al. (2002), despite the simulated source size being larger than that used in the experiments. Dosio and de Arellano (2006) also calculated the statistics of concentration fluctuations in the coordinate system relative to the centre of mass and obtained good qualitative agreement with a gamma PDF.

An accurate investigation on the effect of the grid resolution on the concentration fluctuation statistics (up to the fourth moment) was very recently presented by Ardeshiri et al. (2020) using the open source code parallelized LES model (PALM, Maronga et al. 2015). Notably, by spanning a wide range of grid refinement (the source was resolved from a minimum of one to a maximum of $8^3$ grid cells), Ardeshiri et al. (2020) showed that the dependence of concentration statistics on the grid size is not monotonic and explained the mechanism by which grid resolution affects concentration fluctuations. They also showed that the gamma PDF is an excellent model for concentration fluctuations from point sources, but only for downwind positions beyond the peak of concentration fluctuation intensity.

### RANS Methods

The application of RANS methods to study concentration fluctuations from a steady localized source in a turbulent boundary layer goes back to Csanady (1967), who investigated the closure of the transport equation for the concentration variance, i.e. Eq. 4.

Csanady (1967) used gradient diffusion relationships to close both the turbulent flux of scalar concentration $\left\langle u'_i \phi ' \right\rangle = -K \partial \left\langle \phi \right\rangle /\partial x_i$ and the third-order moment $\left\langle u'_i \phi '^2 \right\rangle = -K_{\phi '^2} \partial \left\langle \phi '^2 \right\rangle /\partial x_i$ (*K* and $K_{\phi ^{'2}}$ are, respectively, the turbulent diffusivities of the mean and variance concentration field). He also proposed to close the scalar dissipation as,

6 $$\begin{aligned} \varepsilon _{\phi } = \frac{\left\langle \phi '^2 \right\rangle }{T_{\phi }}, \end{aligned}$$

where $T_{\phi }$ is a characteristic time scale of scalar dissipation. Csanady (1967) proposed $T_{\phi }$ to be proportional to the mean advective travel time downwind from the source $T_{a}= x/\left\langle u \right\rangle$, where *x* is the downwind distance from the source. With the application of these closures, Eq. 4 becomes

7 $$\begin{aligned} \frac{\partial \left\langle \phi '^2 \right\rangle }{\partial t} + \left\langle u_i \right\rangle \frac{\partial \left\langle \phi '^2 \right\rangle }{\partial x_i} = 2 K \left( \frac{\partial \langle \phi \rangle }{\partial x_i} \right) ^2 + \frac{\partial }{\partial x_i}\left( K_{\phi '^2}\frac{\partial \left\langle \phi '^2 \right\rangle }{\partial x_i}\right) -\frac{\left\langle \phi '^2 \right\rangle }{T_{\phi }}. \end{aligned}$$

He found an analytical solution of Eq. 7 by assuming steady state homogeneous turbulence, slender plume approximation, self similarity, and $K_{\phi '^2}=K$. Kewley (1978) found an alternative analytical solution by assuming a balance between production and dissipation and he firstly demonstrated the mechanism by which off-centreline concentration variance double peaks may be generated in a dispersing plume. Other analytical solutions of simplified formulations of Eq. 7 (Netterville 1979; Wilson et al. 1982b) are discussed in Sect. 4.2.

Other authors (e.g. Lewellen and Teske 1976; El Tahry et al. 1981) presented instead numerical solutions of Eq. 7, considering dispersion within a turbulent boundary layer. Sykes et al. (1984) solved the full set of equations for mean concentration, fluxes, and concentration variance and compared their results to experimental measurements of Fackrell and Robins (1982b). Sykes et al. (1984) emphasized the need of introducing a characteristic scalar length scale to correctly model the concentration variance dissipation rate. Based on Durbin (1980) and Sawford (1982) (see Sect. 3.4 below), they also showed that relative dispersion and inertial range scaling should be used to define the evolving characteristic scalar length scale of the plume as

8 $$\begin{aligned} \frac{d L_\phi }{dt} = \alpha _1 \sigma _{ur}, \end{aligned}$$

where

9 $$\begin{aligned} \sigma _{ur}^2 = \left\langle u'_i u'_i \right\rangle (L_\phi /L)^{(2/3)}, \end{aligned}$$

is the turbulent kinetic energy involved in the relative dispersion process. The constant $\alpha _1$ is empirical, *L* is the characteristic turbulent macroscale related to the size of larger scale eddies. These definitions imply that the dissipation time scale is

10 $$\begin{aligned} T_{\phi }=\alpha _2 L_\phi / \sigma _{ur}, \end{aligned}$$

where $\alpha _2$ is a second empirical constant. According to this formulation, the variance dissipation rate evolves rapidly when the plume is small and slows down while the plume grows. However, Sykes et al. (1984) stressed that Eq. 9 becomes inappropriate for a large travel time, when $L_\phi \gg L$, and they imposed a further relationship $\varepsilon _{\phi }\propto \left( \left\langle u'_i u'_i \right\rangle ^{1/2} / L_\phi \right)$. Thomson (1997) analyzed the Sykes et al. (1984) model in view of his theoretical findings of the behaviour of the scalar dissipation in three asymptotic regimes. In the most relevant regime, Thomson (1997) theory predicts that $T_{\phi }=2 t /(3 \lambda _s)$, where $\lambda _s$ is the source geometrical dimensionality, e.g. $\lambda _s=2$ for a continuous point source, and *t* can be considered to be the travel time ($T_{a}$) in this context. The Thomson (1997) analysis showed that the two constants in the Sykes et al. (1984) model must be related to the source dimensionality so as to be consistent with his theoretical analysis. Some simplifications of the Sykes et al. (1984) model were introduced by other authors. Sykes et al. (1986) obtained a set of ordinary differential equations for the downwind evolution of the integrated quantities over the plume cross-sections and Galperin (1986) simplified the model for the scalar length scale discarding the need to solve a prognostic equation for the length scale but introducing less general assumptions. These early applications, adopting second-order closure models, showed the ability of RANS models to predict concentration variance in neutral stability conditions. To our knowledge, there are no RANS applications that simulate concentration fluctuations in a convective boundary layer. These are indeed expected to be critical for second-order RANS methods, which cannot formally handle counter-gradient turbulent transport typical of convective conditions (e.g. Stull 1988).

In more recent years, RANS methods have been widely applied to simulate concentration fluctuations in urban and urban-like array of obstacles (e.g. Efthimiou 2019). Andronopoulos et al. (2001) applied a second-order closure to forecast concentration variance for an idealized road intersection. As with Csanady (1967), Andronopoulos et al. (2001) used diffusion coefficients, as defined by Bartzis (1989), to close the RANS equations for the mean and variance. The length scale for the scalar dissipation rate was simply assumed to be in equilibrium and equal to the turbulent length scale used in the turbulent closure, i.e. $L_\phi =L$. This implies that the scalar dissipation time scale is proportional to the turbulent time scale (Warhaft and Lumley 1978)

11 $$\begin{aligned} T_{\phi } \propto T (=E/\varepsilon ). \end{aligned}$$

A similar approach was used by Milliez and Carissimo (2008) to simulate the concentration fluctuations measured in the MUST experiment (Biltoft 2001; Yee and Biltoft 2004) for a plume dispersing in an obstacle array. Hsieh et al. (2007) compared the equilibrium approximation with a simplification of Sykes et al. (1984) variable length-scale approach defining $L_\phi \propto (\sigma _y \sigma _z)^{(1/2)}$, with $\sigma _y$ and $\sigma _z$ being the plume crosswind and vertical spreads of the mean plume, respectively. They found that the variable length scale ensures an improved performance in reproducing the MUST experimental results. Yee et al. (2009) further improved the length scale formulation (and related dissipation time) adapting Cassiani et al. (2005a), who formulated a mixing time scale for a Lagrangian PDF micromixing model. It is worth noting that, as discussed in Cassiani et al. (2005a) and outlined in Sect. 3.3, the dissipation time used in the second-order closure model is linked to the micromixing time used in the PDF transport equation. Yee et al. (2009) obtained a remarkably good agreement with the MUST experimental measurements. A different approach was used by Efthimiou and Bartzis (2011) and Efthimiou et al. (2016b); they generalized the proportionality between $T_{\phi }$ and $T_a$ (as originally proposed by e.g. Csanady (1967)) defining a local and non-homogeneous mean travel time from the ratio of the concentration of two chemical species emitted at the source

12 $$\begin{aligned} T_{\phi } \propto T_{travel} \left( =\frac{1}{\lambda _D}\ln \frac{\phi _d}{\phi _c}\right) , \end{aligned}$$

where $\phi _c$ and $\phi _d$ denote the concentration of a passive and decaying scalar, respectively, the latter characterized by a constant decay rate $\lambda _D$.

### PDF Methods

What we refer to as ‘PDF methods’ for the prediction of concentration fluctuations in atmospheric flows is an approach that, historically, arises as the conjunction of two parallel research fields. Firstly, the atmospheric dispersion modelling community, who are devoted to the formulation of Lagrangian one-particle dispersion models, and secondly, to the turbulent combustion community on reacting flows. In what follows our aim is two-fold: (i) to show how these methods have been implemented in atmospheric Lagrangian one-particle dispersion models whose use was initially limited to the prediction of the mean concentration field, and (ii) to briefly review how these methods originated in a broader and more general theoretical framework for calculating turbulent reacting flows based on the formulation of transport equations, in order to forecast the PDF of all the relevant turbulent variables.

Fundamental concepts on Lagrangian one-particle dispersion models that are not strictly linked to the problem of concentration fluctuations (nonetheless needed by the less acquainted reader to understand what follows) are presented in the Appendix. These models are used to simulate the trajectories of independent single fluid marked particles within a turbulent flow. Each of these particles carries an unaltered amount of tracer, so that

13 $$\begin{aligned} \frac{d \phi ^*}{dt} = 0 \end{aligned}$$

where, as in the Appendix, we use the star to denote a particle quantity. These models are not suited to the estimate of higher-order (than the mean) concentration statistics, since they are unable to simulate any mixing process. In their basic application, Lagrangian one-particle models are therefore devoted to the estimate of the mean concentration only, and consider only the marked particles passing through the source. In their simpler form of random displacement models (see Eq. 38 in the Appendix) they use the gradient diffusion approximation. Application of this approach to the estimates of fluctuation statistics requires instead to fill the whole domain with particles (e.g. Cassiani et al. 2005a, b), each of them moving according to Eq. 38. This allows for the inclusion of the dissipative mixing process in the simulation and therefore the estimate of the concentration PDF. Yet, this necessitates a further equation for the particle concentration state to be solved,

14 $$\begin{aligned} d\phi ^* = {\varOmega }dt \end{aligned}$$

where ${\varOmega }$ denotes a generic mixing model, i.e. a term that allows each particle to exchange scalar concentration with the surrounding particles. Several types of mixing models have been proposed in the literature. The simplest model is the interaction by exchange with the mean (IEM), which has been used for decades in the context of turbulent combustion (Dopazo and O’Brien 1974; Pope 2000),

15 $$\begin{aligned} {\varOmega }= -\frac{(\phi ^* - \left\langle \phi \right\rangle )}{\tau _m}, \end{aligned}$$

where $\tau _m$ is the mixing time scale (see Sect. 3.3.1). The IEM model uses a simple relaxation of the local concentration towards a local mean, but it has been shown to introduce spurious fluxes altering the mean concentration field (Pope 1998; Sawford 2004; Cassiani et al. 2007b; Viswanathan and Pope 2008). Despite this shortcoming, the IEM model has been widely used, for example, by Dixon and Tomlin (2007) to simulate fluctuations in an idealized urban street canyon and by Cassiani et al. (2010) to simulate the effects of SGS emission heterogeneity in a mesoscale dispersion model.

In a Lagrangian particle model including micromixing, the concentration moments can be simply computed by using, for example, a cell average

16 $$\begin{aligned} \left\langle \phi ^m \right\rangle _j = \frac{\sum _{i=1}^{N_j} \phi ^{*m}_i}{N_j} , \end{aligned}$$

where *i* indicates a particle, $N_j$ is the number of particles within the *j*th grid cell, and $\left\langle \phi ^m \right\rangle _j$ is the *m*th order concentration moment in the cell. These calculated moments are affected by statistical noise and the error decreases by increasing the number of simulated particles (see e.g. Cassiani et al. 2007b). The concentration PDF can also be estimated from the particle concentrations (see e.g. Pope 1985), with different methods, including the simple box counting.

Assuming that the particle are uniformly distributed in the domain, it can be shown (see e.g. Pope 2000), that Eqs. 38 and 14 with the definition in Eq. 15, correspond to a transport equation for the concentration PDF of the form

17 $$\begin{aligned} \frac{\partial f_\phi }{\partial t} + \left\langle u_i \right\rangle \frac{\partial f_\phi }{\partial x_i}= \frac{\partial }{\partial x_i} \left( K \frac{\partial f_\phi }{\partial x_i}\right) + \frac{\partial }{\partial \psi } \left[ f_\phi \frac{(\psi - \left\langle \phi \right\rangle )}{\tau _m}\right] . \end{aligned}$$

A comparison between Eqs. 17 and 5 shows that the turbulent flux of probability has been closed by a standard gradient-diffusion approach,

18 $$\begin{aligned} - \frac{\partial }{\partial x_i} \left( f_{\phi } \left\langle u_i' \Big | \psi \right\rangle \right) =\frac{\partial }{\partial x_i} \left( K \frac{\partial f_\phi }{\partial x_i}\right) , \end{aligned}$$

while the conditional Laplacian (containing all information about the scalar fluctuation dissipation) has been closed by the IEM model (see e.g. Pope 2000)

19 $$\begin{aligned} \left\langle D\nabla ^2\phi | \psi \right\rangle = -\frac{(\psi - \left\langle \phi \right\rangle )}{\tau _m}. \end{aligned}$$

Bertagni et al. (2019) have recently found a formal solution for the statistical moments of concentration from the transport equation of the PDF (Eq. 17). In particular, they derived an analytical relation for the passive scalar variance $\sigma _\phi$, which does not require a numerical or empirical approach and, encouragingly, it has been shown to well resemble wind-tunnel data from a point source in a neutral boundary layer.

In recent years, most commonly, the random displacement model has been replaced in atmospheric applications by stochastic equations for the position and velocity of particles (Eqs. 39 and 40 in the Appendix). Similarly to that explained above for the random displacement model, the physical state described by Eqs. 39 and 40 can be augmented with Eq. 14 for the concentration. If the particles are uniformly distributed in the domain of interest, it can be demonstrated (see e.g., Pope 2000) that this system of stochastic differential equations corresponds to the following transport equation for the joint velocity–scalar concentration PDF (e.g. Cassiani et al. 2005a, 2007b)

20 $$\begin{aligned} \frac{\partial f_{\phi u} }{\partial t} + v_i \frac{\partial f_{\phi u}}{\partial x_i} =-\frac{\partial }{\partial v_i} \left[ a_i f_{\phi u} \right] + \frac{\partial ^2}{\partial v_i \partial v_j} \left[ B_{ij} f_{\phi u} \right] - \frac{\partial }{\partial \psi } \left[ f_{\phi u} {\varOmega }(\psi ) \right] , \end{aligned}$$

where $\psi$ is the sample space variable of the concentration $\phi$, and $v_i$ is here used to denote the sample space variable of the (random variable) velocity, $u_i$. The symbol ${\varOmega }(\psi )$ is used here to indicate a generic deterministic mixing model, noting that $B_{ij}=b_{ik}b_{jk}/2$ (see Eq. 42 in the Appendix). We briefly note that in flows with a variable air density the requirement of uniform particle distribution must be replaced by particles distributed according to the air density (Thomson 1987; Cassiani et al. 2015).

The use of the joint PDF $(f_{\phi u})$ allows the introduction of conditional (over velocity) averages. By replacing the unconditional mean concentration in the IEM model with the conditional mean, one obtains the interaction by exchange with the conditional mean (IECM) micromixing model,

21 $$\begin{aligned} {\varOmega }=- \frac{(\psi -\langle \phi |{\varvec{v}} \rangle )}{\tau _m}, \end{aligned}$$

introduced by Fox (1994) and Pope (1998). Pope (1998) and Sawford (2004) discuss why the IECM model does not create spurious fluxes and does not alter the mean concentration field. Sawford (2004) also obtained good agreement for mean and higher-order moments of concentration measured in decaying grid turbulence. The IECM model was firstly used for modelling concentration fluctuations from point and line sources in atmospheric-like boundary layers under neutral stability conditions by Cassiani et al. (2005a) and in convective stability conditions by Cassiani et al. (2005b) and Luhar and Sawford (2005). Cassiani et al. (2007b) applied the IECM model to simulate concentration fluctuations from a line source in canopy-generated turbulence, and Leuzzi et al. (2012) used it to simulate the fluctuations of a plume dispersing through an array of obstacles in the MUST experiment.

An interesting aspect of the IECM model has been discussed by Sawford (2004), who demonstrated that in homogeneous turbulence in the limit of $\tau _m \rightarrow 0$, i.e., when the conditional average fully determines fluctuations, the IECM model is equivalent to a simple meandering plume model where a particularly simple form of the two-point velocity correlation is assumed in the relative expansion (Sawford 2004; Cassiani et al. 2005a).

Both the IEM and IECM modelling approaches discussed above suffer from the fact that they do not allow relaxation of the PDF shape in the absence of mean scalar gradients (e.g. Pope 2000, p. 550). This issue may significantly alter the forecast high-order concentration moments of a dispersing plume, as was shown by Marro et al. (2018). Other approaches to close the micromixing term include mapping closures (Pope 1991), Curl’s models (Curl 1963; Hsu and Chen 1991), the Euclidean minimum spanning tree model (Subramaniam and Pope 1998), stochastic models based on the Langevin (Valiño and Dopazo 1991; Pozorski and Minier 1998; Heinz 2003) or the Fokker–Planck (Fox 1994) equations, and models that directly require spectral information (Vaithianathan et al. 2002). Recently, Meyer and Jenny (2013) investigated the properties of the velocity-conditioned Curl’s model and proposed a new velocity-conditioned mixing model that proved to be computationally efficient and to have better properties compared to the IECM model with respect to the ability to correctly relax PDF shape.

So far we have discussed how PDF methods used to forecast the moments and PDF of the concentration have been introduced in the atmospheric community as an extension of Lagrangian single-particle dispersion models. However, PDF methods have a broader application and aim to solve transport equations for the PDF of several flow properties. Historically, the PDF methods originated from the seminal work of Lundgren (1967), who first recognized that a hierarchy of unclosed transport equations for the velocity PDF can be obtained from the Navier–Stokes equations by using the properties of the fine grained PDF. More generally, the PDF can be used a single or several flow statistical properties, and for single or multiple points, and single or multiple times (e.g., Dopazo 1994). In what we have discussed so far, we limited our review to PDF methods to forecast the one-point one-time PDF for the concentration, and the PDF in the transport equations was intended as the concentration distribution or at most the velocity–concentration joint distribution. Yet, other turbulent variables can be included. One of the most remarkable points in favour of the use of the PDF approach is that chemical reactions of any order appear in closed form. Indeed, previous reviews on PDF methods have mainly focused on the simulation of reacting and dynamically-active scalars with emphasis on combustion processes (O’Brien 1980; Pope 1985; Dopazo 1994; Haworth 2010). PDF methods are also treated in books about turbulent reacting flows (Kuznetsov and Sabel’Nikov 1986; Pope 2000; Fox 2003; Heinz 2003; Haworth and Pope 2011).

Here, the review focuses on atmospheric applications and as customary (e.g., Thomson 1987) we assumed that the one-point one-time velocity PDF has a known analytical shape. Therefore, we do not discuss in any detail the use of PDF methods to forecast the velocity field (e.g., Pope 1994, 2000), despite its interest when dealing with air pollution problems (see for example the work by Bakosi et al. (2009) who simulated dispersion in an urban canyon). Indeed, the computational requirement to forecast the velocity PDF in atmospheric domains is prohibitive. In single-time single-point PDF methods, similarly to RANS methods, the information about the mean turbulent time and length scales must be parametrized based on known quantity or alternatively a turbulent frequency variable could be included in the joint PDF (Pope 2000; Duman et al. 2016).

The PDF transport equations are multidimensional. Any added scalar is a further dimension in the equation and, in case of joint velocity scalar PDF equations, any velocity component adds a further dimensions to the domain. For example if the joint PDF $f_{\phi u}$ was considered in a 3D domain, Eq. 20 would have seven dimensions. We emphasize that even when calculating the concentration PDF $f_\phi$, based on an assumed mean velocity and PDF $f_u$, the PDF transport equation must be solved for the specific initial and boundary conditions (Cassiani et al. 2005a, b, 2007b) and the equation is still in seven dimensions. Due to the high dimensionality, stochastic approaches are commonly the preferred methods to numerically solve the PDF transport equation. The approaches can be Eulerian stochastic field methods (Valiño 1998; Sabel’nikov and Soulard 2005; Garmory et al. 2006; Cassiani et al. 2010; Wang et al. 2018) or more commonly the Lagrangian particle-mesh methods (Pope 2000; Fox 2003; Heinz 2003), which has been briefly outlined above for the special case of the calculation of the concentration PDF by extending atmospheric Lagrangian one-particle dispersion models.

For atmospheric dispersion applications, assuming that the velocity PDF is known, considerable optimization in the calculations is possible. In the context of Lagrangian particle-mesh algorithm, Cassiani et al. (2005a, (2005b) proposed the use of expanding grid to model plume dispersion, and Cassiani et al. (2007b) the use of nested grid with straightforward particle splitting and erasing procedures. These algorithms advance particles in parallel and allow the straightforward inclusion of chemical processes and therefore the possibility to model fluctuations of reacting plumes. Algorithms advancing particles in parallel allow also the use of mixing algorithm based on direct particle interaction such as Curl and modified Curl methods (Meyer and Jenny 2013). If there is no ambition to consider chemically reactive species, and by using only mixing algorithm based on the mean concentration (i.e., IEM and IECM), the assumption of a known velocity PDF allows also the pre-calculation of the mean values to be used in mixing models. This approach permits the use of simple algorithms amenable of trivial parallelization where each particle is independently advanced (Luhar and Sawford 2005; Postma et al. 2011a, b). This simplified approach does not allow the use of mixing algorithm based on particle interaction nor the inclusion of chemical reactions. Moreover, if the simpler IEM algorithm is used with a pre-calculated mean value an inconsistency arises because, as discussed above, the IEM model creates spurious fluxes that tend to alter the mean value from the pre-calculated (and correct) mean. This inconsistency must be considered when evaluating the fluctuations.

PDF transport equation methods can also be coupled to LES to provide what is perhaps the most advanced way of simulating concentration fluctuations. This approach is named filtered density function (Colucci et al. 1998). Aguirre et al. (2006) used this coupled approach for simulating a turbulent reacting plume. Cassiani et al. (2007a) used this approach to simulate the concentration PDF generated by a scalar source under-resolved by the LES.

#### The Micromixing Time Scale

The mixing, or micromixing, time scale $\tau _m$ is a key quantity in modelling the dissipative effect of molecular diffusion on fluctuations. In the idealized case of homogeneous turbulent mixing (with no mean scalar gradient), the mixing time scale is equal to the dissipation time scale of concentration variance, i.e. $\tau _m=T_\phi$. In this case, the initial mixing time scale is imposed by the initial correlation length scale of the scalar field. If the correlation length scale is initially smaller compared the turbulent length scale it will increase, eventually reaching that of the turbulent velocity length scale (Sreenivasan et al. 1980). The increase in the scalar length scale will also increase the mixing and dissipation time scales until an equilibrium is reached. At the equilibrium, the mixing time scale is only imposed by the velocity statistics and has therefore to be proportional to the turbulent time scale *T*. However, when considering the condition of inhomogeneous mixing, like the dispersion from a localized release, the dissipation of fluctuations and, therefore, the parametrizations of $\tau _m$ and $T_{\phi }$ depend also on the source dimensionality (line, point) and size (e.g., Thomson 1996, 1997; Cassiani et al. 2005a). Nonetheless, the proportionality between turbulent and dissipation (or mixing) time scale has been often used also for these conditions, but the constant of proportionality varies widely depending on the specific model or experimental set-up. We note that the behaviour of the dissipation time scale for a localized source is similar to that of homogeneous random field starting with a correlation length scale smaller than the turbulence scale, if the former is integrated across the plume (Thomson 1996)

Additionally, the value of $\tau _m$ also depends on the the mixing model used (conditioned on the velocity or not). As discussed in Cassiani et al. (2005a), for the IECM model ${\tau _m}=T_\phi (1- \langle \langle \phi ' | {\varvec{u}} \rangle ^2 \rangle /\sigma _\phi ^2)$, while for the IEM model $\tau _m=T_\phi$. Sawford (2004) suggested that, for a localized source, $\tau _m$ is closely related to the process of relative dispersion, in agreement with previous considerations on $T_\phi$ made by Sykes et al. (1984). The link between relative dispersion and scalar dissipation has been formally demonstrated for the special case of homogeneous turbulent mixing by Thomson (1996) (see also the discussion in Sawford (2001)). Based on this physical link Cassiani et al. (2005a) expressed the mixing time as $\tau _m\ \propto \sigma _r/\sigma _{\varvec{u}r}$, where $\sigma _{\varvec{u}r}$ is the relative (to the local centre of mass) velocity standard deviation and $\sigma _r$ the relative spread standard deviation, expressed as

22 $$\begin{aligned} \sigma _r^2=\frac{d_r^2}{1+(d_r^2-d_0^2)/(d_0^2+ (4/3) E \, T_L t)}, \end{aligned}$$

where $d_0$ is the source size, $T_L$ the Lagrangian integral time scale, *t* the travel time of a Lagrangian particle, and $d_r^2$ the inertial range relative dispersion spread, parametrized as

23 $$\begin{aligned} d_r^2=C_r \varepsilon (t_0+t)^3, \end{aligned}$$

where $C_r$ is the Richardson-Obukhov constant (e.g., Monin and Yaglom 1975; Franzese and Cassiani 2007) and $t_0$ a characteristic time of the source size. Here, $\sigma _{\varvec{u}r}^2$ is parametrized as $\sigma _{\varvec{u}r}^2= \left( 2/3\right) E (\sigma _r/L)^{(2/3)}$, if $\sigma _r < L$, while if $\sigma _r > L$ the bounding value $\sigma _{\varvec{u}r}^2 = \left( 2/3\right) E$ is imposed; *L* is the characteristic turbulent length scale and depends on the stability conditions. Under neutral stability $L=E^{\left( 3/2\right) }/\varepsilon$, while in convective conditions $L=h_b$, where $h_b$ is the depth of the convective boundary layer (Cassiani et al. 2005b). Note that this model for $\tau _m$ is similar to that of Sykes et al. (1984) for $T_\phi$, but more consistent with both the inertial range scaling argument and the Taylor’s kinematic dispersion theory. The empirical proportionality constant in $\tau _m\ \propto \sigma _r/\sigma _{\varvec{u}r}$ was chosen to account for source dimensionality.

In non homogeneous turbulence, Eq. 23 is discretized to capture the inhomogeneity along a particle trajectory (Cassiani et al. 2005a). In Cassiani et al. (2007b), an upper bound to the resulting time $\tau _m$ was set when this is larger than its equilibrium value proportional to the turbulent time scale *T*. This formulation (or a derived one) has been also applied to PDF (RANS) simulations of concentration fluctuations in urban canopies (Yee et al. 2009; Leuzzi et al. 2012). Recently, Bertagni et al. (2019) applied it to an analytical solution for the second-order statistical moment of concentration in a neutral boundary layer. Yet, more research on a general definition of $\tau _m$ remains crucial to improve the generality of the mixing models.

### Lagrangian Two-Marked-Particles Methods for Concentration Variance

Another possible use of the Lagrangian marked-particles framework to estimate concentration fluctuations statistics is provided by the two-marked-particles methods. These methods rely on the same concept of the one-particle models, i.e. that of writing the relationship between the motion of the marked particles and the moments of the scalar concentration. As discussed in the Appendix, Eq. 37 shows the relation between the mean concentration and the one-particle transition probability density function. This relationship can be extended to second-order moments by considering the motion of two correlated particles (below indexed *A* and *B*) (see, e.g., Thomson 1990; Sawford 2001)

24 $$\begin{aligned} \left\langle \phi ^{A} \phi ^{B} \right\rangle = \iint _{t_0^{A},t_0^{B}} \iint _{V} p^{A,B} S^{A} S^{B} d\varvec{x}_0^A d\varvec{x}_0^B dt_0^A dt_0^B , \end{aligned}$$

where $\phi ^A=\phi (\varvec{x}^A,t^A)$, $S^A=S(\varvec{x}_0^A,t_0^A)$, (equivalent notation for the particle *B*). $p^{A,B}=p(\varvec{x}^{A},\varvec{x}^{B},t^{A},t^{B};\varvec{x}^{A}_0,\varvec{x}^{B}_0,t_0^{A},t_0^{B})$ is the probability that two particles originally at position $\varvec{x}_0^{A}$, $\varvec{x}_0^{B}$ at time $t_0^{A}$, $t_0^{B}$ will end their trajectory in $\varvec{x}^A,\varvec{x}^B$ at time $t^A,t^B$. By taking $t^A=t^B=t$ and $\varvec{x}^A=\varvec{x}^B=\varvec{x}$, the relationship in Eq. 24 describes the second-order moment of concentration in a single point and time, and can be used to compute the concentration variance in conjunction to Eq. 37. Equation 24 implies, through $p^{A,B}$, that is possible to model realizations of the correlated motion of two particles. We note that often the equations above are written in backward formulation so that the initial time correspond to the sampling time. This reversed formulation has many practical advantages especially if the concentration fluctuations need to be calculated only in few specific points (e.g. Sawford 2001). The formulations above can also be extended to *n*th order concentration moment but this implies the ability to model the correlated motion of *N* particles. Egbert and Baker (1984) and Sawford (2001) explained why a marked particle can be used to simulate the evolution of concentration variance, and therefore its dissipation, even if the molecular diffusivity is not explicitly included. Summarizing, this goes back to the fact that, at high Reynolds and finite Schmidt number, the actual rate of dissipation of scalar fluctuations is independent from molecular diffusivity. This is recognized also by the parametrization presented above for RANS models (Eqs. 6 and 10) and models PDF (see Sect. 3.3.1), where it is clear that dissipation is driven by turbulent scales. Sawford (2001) discussed in detail the implication of this in terms of motion of marked particles and marked molecules, with the latter explicitly including molecular diffusivity. In a marked Lagrangian framework, neglecting molecular diffusivity corresponds to the assumption of an infinite Reynolds number with an inertial subrange extending to arbitrarily small time and space scales. These arguments were already discussed in Durbin (1980) and Thomson (1990). An important point to recognize is that the zero molecular diffusivity limit is singular and, if properly handled, does not imply zero dissipation (Sawford 2001).

Thomson (1990) used the Fokker–Planck equation of the Markov process for two-particle velocity and position to show a general approach by which, assuming a known analytical shape for the two-point velocity PDF, it is possible to formulate a consistent (well-mixed compliant) Lagrangian stochastic model for the correlated motion of two particles. The general form of the Lagrangian equations for the velocity and positions of two particles is the same of Eqs. 39 and 40 for a single particle but with the index extending between one and six, indices 1–3 related to particle one and indices 4–6 to particle two. The definition of the diffusion coefficient for the Thomson (1990) two-particle model follows exactly that of the one particle model, Eq. 42 in the Appendix. The diffusion coefficient represents the part of the acceleration that is uncorrelated from one time to the next and it is therefore reasonable to assume it independent of any property of the second particle (Thomson 1990). The definition of the drift coefficient, ensuring the respect of well-mixed conditions, follows the same methods used by Thomson (1987) for one-particle models (briefly discussed in the Appendix) but with several complications. However, the details of the formulation goes beyond the scope of the present review.

Despite their physical appeal, the adoption of two-particle models have been so far mainly limited to dispersion in homogeneous isotropic turbulence due to the difficulty in formulating these models in non-homogeneous non-isotropic turbulence. Indeed, these methods have been very rarely used to calculate concentration fluctuations in more complex flows for operational purposes. Exceptions are the works by Kaplan and Dinar (1993) and Cohen and Reynolds (2000). In simulating the dispersion from a line source in a canopy flows, Cohen and Reynolds (2000) simplified the approach assuming a trivial form for the two-particle correlation (where particles are initially correlated and subsequently move independently). This simple correlation model was named the NGLS model by Thomson (1990) since it is rooted in Novikov (1963), Gifford (1982), and Lee and Stone (1983). Despite this strong assumption, results by Cohen and Reynolds (2000) showed a satisfactory agreement with the experimental results by Raupach and Legg (1983). The model proposed by Kaplan and Dinar (1993) is rather general but uses the assumption that the two-particle correlation is entailed in the diffusion term of the stochastic equations (see Eq. 40 in the Appendix). As mentioned above, this coefficient should represent the part of the acceleration that is uncorrelated from one time to the next ( Thomson 1990) and it seems in contrast with the physical intuition that the part of the acceleration that is uncorrelated from one time to the next is correlated with that of a second particle, which can be further away. More recently Weil et al. (2018) proposed the use of a two-particle model, based on Thomson (1990) approach, in connection to LES.

### Lagrangian Stochastic Heuristic Methods

Recently, some authors implemented heuristic extensions of Lagrangian one-particle methods to include the ability to forecast concentration variance. The main reason for these developments is retaining the flexibility and computational efficiency of Lagrangian single-particle methods. In fact, we remind that in a Lagrangian single-particle method only particles passing through the scalar source are modelled, while in a Lagrangian solver of a PDF transport equation the whole volume of interest inclusive of the “background” needs to be modelled (i.e, filled with computational particles). However, in a single-marked-particle framework, the information about mixing and variance dissipation are not naturally included.

Cassiani (2013) proposed to assign to each (*i*th) released particle (from the source location) a further state variable for the particle volume $vp^*_i$ and a conserved mass $m^*_i$, according to the marked-particle concept, for this reason the model is named volumetric particle approach (VPA). From volume and mass, a concentration can be defined and an IEM micromixing model (see Eq. 15), borrowed from what is done in PDF transport equation methods, is used to compute the relaxation of the particle concentration towards the local mean value. The mean value necessary for the relaxation is computed by discretizing the domain in grid cells of volume $VC_j$. From the updated concentration an updated volume can be computed, i.e. the particle volume would increase due to mixing. It is worth noting that $vp^*_i$ does not represent the real volume of the particles, but it is a variable which increase is meant to represent the homogenization of the concentration due to the scalar dissipation process. The expression for the second-order concentration moments in the VPA approach is

25 $$\begin{aligned} \left\langle \phi ^2 \right\rangle _j = \sum _{i=1}^{N_j} \phi ^{*2}_i \frac{vp^*_i}{VC_j} \end{aligned}$$

where $\phi ^*_i$ is the concentration of the *i*th particle and $N_j$ the number of particles within the cell *j*. As evidenced by Cassiani (2013), this model implies an extremely simplified (and unrealistic) form for the concentration PDF, where a fraction of the volume within each grid cell is at constant background state (e.g., zero) and the rest of the volume is at the particle concentrations

26 $$\begin{aligned} f(\psi )=\delta (\psi )\left( 1- \frac{\sum _{i=1}^{N_j} vp^*_i }{VC_j}\right) + \sum _{i=1}^{N_j} \delta (\psi - \phi ^*_i) \frac{vp^*_i}{VC_j} \end{aligned}$$

where $\psi$ is the sample space variable. Due to this simplified representation of the PDF only the first- and second-order moments of concentration can be predicted by the model, while higher-order moments are generally incorrect. A micromixing time, similarly to what formulated for the IEM model in PDF transport equation methods, is used to ensure the correct dissipation rate for the concentration fluctuations. Cassiani (2013) found that concentration variance predictions were in satisfactory agreement with experiments in neutral boundary layer, canopy turbulence, and decaying grid turbulence ( Fackrell and Robins 1982b; Raupach and Legg 1983; Brown and Bilger 1998). Marro et al. (2018) used the model of Cassiani (2013) to simulate the measurements of Nironi et al. (2015). Furthermore, they showed that a gamma PDF, fully defined by the first two moments, ensures a satisfactory agreement for the skewness and the kurtosis. Marro et al. (2018) named this approach volumetric particle gamma model.

Manor (2014) noted that, by using gradient-diffusion approximations, the closed concentration variance equation is similar to a standard mean advection–diffusion equation. Therefore, he proposed to use the same Lagrangian stochastic one-particle methods used for the mean concentration to solve this equation. We note that this concept is similar to the earlier approach by Netterville (1979) and Wilson et al. (1982a), who obtained Gaussian analytical solutions for the concentration variance equation, see Sect. 4.2. Manor (2014) closed the diffusion coefficient by using the apparent eddy-diffusivity concept, $K_{u,v,w} = 2 \sigma ^2_{u,v,w} T_{L{u,v,w}}$, thus using the same flow variables used to formulate a one-particle Lagrangian stochastic model. With this closure, the variance production term in Eq. 7 becomes a source term (always positive) depending only on the mean concentration field,

27 $$\begin{aligned} -2 \left\langle u'_i \phi ' \right\rangle \frac{\partial \left\langle \phi \right\rangle }{\partial x_i} = 2 \sigma _{ui}^2 T_{Li} \left( \frac{\partial \left\langle \phi \right\rangle }{\partial x_i} \right) ^2, \end{aligned}$$

where no summation is implied in the first two indexes on the right-hand side. This term can be pre-calculated, and assigned to modelled particles, depending on their positions in the domain. The effects of scalar dissipation are modelled using an additional state variable for the particles, the carried amount of concentration variance, $\sigma _{\phi p}$, and allowing it to decay with a dissipation time scale $T_{\phi }$. A simple model of the form $T_{\phi } \propto T_L$ is used. As extensively discussed above in Sects. 3.2 and 3.3.1 this type of definition is valid only when the scalar length scale is larger than the turbulent integral length scale. Manor (2014) applied the model to dispersion in a urban area as measured in the JU2003 field experiment. Ferrero et al. (2017) applied Manor (2014) approach to the experimental measurement of Fackrell and Robins (1982b) by using a dissipation time scale linearly growing in time. Oettl and Ferrero (2017) implemented a similar model in an operational Lagrangian dispersion model for odour impact evaluation.

Kaplan (2014) used the relationships between two-particles models and second-order moment of concentration, Eq. 24, and demonstrated the following relationship to hold

28 $$\begin{aligned} \left\langle \phi (\varvec{x},t)^2 \right\rangle = \int _{V} p(\varvec{x},t;\varvec{x}_0,0) S(\varvec{x}_0,0) {\varPhi }_c(\varvec{x},t;\varvec{x}_0,0) d\varvec{x}_0 , \end{aligned}$$

where *S* is the scalar source function, and $p(\varvec{x},t;\varvec{x_0},0)$ is the probability of a single particle being in $\varvec{x}_0$ at the initial time to be in $\varvec{x}$ at time *t*. The quantity ${\varPhi }_c(\varvec{x},t;\varvec{x}_0,0)$ is defined in Kaplan (2014) and called the conditional-averaged scalar concentration. Here we briefly note that the conditional probability defining this conditional average is the ratio between the two-particle transition probability (see Sect. 3.4) and a one-particle transition probability associated to one of the two particles composing the particle pair (see Sect. 6). To go forward Kaplan (2014) took an heuristic approach and noted that ${\varPhi }_c(\varvec{x}_0,0;\varvec{x}_0,0) = S(\varvec{x}_0,0)$ at the initial time and that for larger time, when two particles are far away, ${\varPhi }_c(\varvec{x},t;\varvec{x}_0,0) = \left\langle \phi (\varvec{x},t) \right\rangle$. Therefore, ${\varPhi }_c(\varvec{x},t;\varvec{x}_0,0)$ is included as an additional state variable transported by the particle and its evolution is again modelled by a simple linear relaxation towards the mean state (i.e., an IEM model) governed by a time scale. Kaplan (2014) used the formulation of Sykes et al. (1984) to model this time scale. Once the model is discretized, the expression used to calculate the second-order concentration moment in a cell of volume $VC_j$ in this approach is

29 $$\begin{aligned} \left\langle \phi ^2 \right\rangle _j = \sum _{i=1}^{N_j} {\varPhi }_{ci}^{*} \frac{m^*_i}{VC_j} , \end{aligned}$$

It is worth noting that this formula can be recast in a form equivalent to that used in the VPA approach previously proposed by Cassiani (2013), since $m^*_i=\phi _i^{*}\times vp^*_i$ and realizing that $\phi _i^*={\varPhi }_{ci}^{*}$, because both are evolved starting from the initial source concentration towards a local mean (calculated in a cell of volume $VC_j$) by the IEM model. This is also discussed in a recent review by Ferrero et al. (2020).

## Phenomenologically-Based Approaches

The models presented in the previous section were all based on the balance equation of a given statistical quantity, characterizing the one-point fluctuations of the scalar concentration. In this balance equation, the statistics of the velocity field appear explicitly and can be provided as an input parameter. In what follows, we review another typology of models whose formulation shortcuts any sort of transport equation, and therefore we have to rely on some empirical evidence. The focus of these models, which we have referred to here as ‘phenomenologically based’, is directly on the the one-point statistics of the scalar concentration. In all of these models (except for the Lagrangian stochastic meandering approach, Sect. 4.1) the information on the statistics of the velocity fields appear only implicitly, i.e. through the determination of parameters modelling the dispersion and the mixing of the scalar.

### Fluctuating Plume Models

The basis of this approach was initially proposed by Gifford (1959) and consists in considering two independent processes in the dispersion of a slender fluctuating plume: the meandering and the relative dispersion (see Fig. 1).

These two mechanisms can be treated as independent with the assumption that they are related to spatial scales well separated (Hanna 1986). Physically, this assumption has to rely on the existence of a spectral gap between the large and the small scale eddies acting on the plume (the reader is referred to the discussion chaired by S. Corssin between F. Gifford and G.K. Batchelor in the last section of Gifford (1959)). This condition is expected to hold only in the very near-field or in the far-field (Yee et al. 1994b).

Mathematically, this assumption permits to express the one-point concentration $\phi$ as a random variable that is function of two independent random variables: the crosswind position of the center of mass due to the meandering $(y_m, z_m)$, and the scalar concentration in a relative frame attached to this centre of mass $\phi _{r}$. These random variables are characterized by specific PDFs, referred to as $f_m$ and $f_{\phi r}$, respectively for position of the centre of mass and concentration in relative frame. Consequently, the PDF of the concentration $\phi$, $f_\phi$, will be the convolution of $f_m$ (the PDF of the location of the plume instantaneous centroid) and $f_{\phi r}$ (the PDF of the concentration field in the centre of mass reference $({x, y_m,z_m}$))

30 $$\begin{aligned} f_\phi \left( \psi ; \varvec{x} \right) = \int _0^{h_b} \int _{-\infty }^\infty f_{\phi r}\left( \psi ;\varvec{x},y_m,z_m\right) f_m \left( y_m,z_m;x\right) dy_m dz_m, \end{aligned}$$

where $$\psi $$ is the sample space variable for the concentration $$\phi $$, while to simplify the notation, we used $$(y_m, z_m) $$ to denote both random variables and sample space variables. The reader should note that, although mean wind shear can be included in this framework, the plume meandering is strictly defined as a crosswind phenomenon. This means that along-wind turbulent dispersion is neglected and therefore, only $$y_m$$ and $$z_m$$ appear in this relationship. Here we used the notation $$f_\phi \left( \psi ; \varvec{x} \right) $$ as customary in the fluctuating plume community, but we stress that this is just the one-point one-time PDF $$f_\phi $$ in the specific point $$\varvec{x}$$.

The assumed independence between meandering and relative dispersion processes implies that the total crosswind plume standard deviations $\sigma _{y,z}$ in absolute coordinates, can be expressed as a function of the outer (meandering, $\sigma _{ym,zm}$) and the inner (relative dispersion, $\sigma _{yr,zr}$) crosswind plume standard deviations. Applying the parallel axis theorem of moments of inertia, one obtains (Csanady 1973; Gailis et al. 2007)

31 $$\begin{aligned} \sigma ^2_{y,z} = \sigma _{ym,zm}^2 + \sigma _{yr,zr}^2. \end{aligned}$$

The quantities $\sigma _{zm}^2/\sigma _{zr}^2$ and $\sigma _{ym}^2/\sigma _{yr}^2$ are usually referred as meander ratios. Two out of the three terms in Eq. 31 are normally parametrized. For instance, Fackrell and Robins (1982b) used the results presented in Hay and Pasquill (1959) and Smith and Hay (1961). Other authors (e.g., Marro et al. 2015) modelled $\sigma _{y,z}$ by applying Taylor’s statistical theory of diffusion (Taylor 1922) and $\sigma _{yr,zr}$ using the Richardson–Obukhov law (Richardson and Walker 1926; Obukhov 1941; Franzese and Cassiani 2007).

In the literature, only few models for the PDF of the centroid location have been used. The main assumption is the statistical independence between plume meandering in the crosswind and vertical directions. In this case, $f_m$ can be expressed as the product of the two components, i.e. $f_m= f_{ym} f_{zm}$, which are functions of the local crosswind spreads, $\sigma _{ym}$ for the horizontal and $\sigma _{zm}$ for the vertical (e.g., Gailis et al. 2007; Marro et al. 2015). Yee and Wilson (2000) showed that the further hypothesis of isotropic dispersion induces the circular symmetry of $f_m$ that, therefore, can be suitably modelled with a normal distribution.

Luhar et al. (2000) proposed a particle-based meandering approach that consists of computing $f_m$ by means of simulations of the centroid trajectories with a Lagrangian stochastic model. This model is especially suitable for problems where analytical tractability of the mean absolute dispersion is not feasible. The approach was used for modelling fluctuations of concentration in a convective boundary layer (Luhar et al. 2000; Franzese 2003) and in a plant canopy by Mortarini et al. (2009), although in this latter case the fluctuations close to the ground could not be well reproduced by the model. This approach requires to parametrize only the relative dispersion spread $\sigma _{yr,zr}$.

The first applications of the fluctuating plume model neglected the concentration fluctuations due to the relative dispersion and only considered the high-order statistics of the concentration due to meandering (Gifford 1959). This approach approximates $f_{\phi r}$ as a Dirac delta distribution $\delta$

32 $$\begin{aligned} f_{\phi r}\left( \psi ; \varvec{x},y_m,z_m\right) = \delta \left( \psi - \left\langle {\phi }_r \left( \varvec{x},y_m,z_m\right) \right\rangle \right) , \end{aligned}$$

where $\left\langle {\phi }_r \right\rangle$ is the spatial distribution of the mean concentration relative to the instantaneous plume centroid. The approximation of $f_{\phi r}$ as a Dirac delta function was implicit in Gifford (1959), whereas it was explicitly discussed in Sawford and Stapountzis (1986).

The relative mean concentration $\left\langle {\phi }_r \right\rangle$ is often parametrized as a Gaussian distribution (e.g., Sawford and Stapountzis 1986; Yee et al. 1994b). However, Marro et al. (2015) approximated it with a double Gaussian in order to take into account the ground reflection, Gifford (1970) used a top-hat distribution, and several authors used a skewed sum of two Gaussian distributions for convective conditions (Luhar et al. 2000; Cassiani and Giostra 2002b; Franzese 2003).

The classic formulation in Eq. 32, neglecting the effects of concentration fluctuations in relative dispersion coordinates, was applied in some works prior to 1994. Gifford (1959) compared the solutions computed through an isotropic two-dimensional model with few experimental data available at that time (Lowry et al. 1951; Gosline 1952) and also with some measurements coming from personal communications. Fackrell and Robins (1982b) simulated the effects on fluctuation intensity of varying the source size in an anisotropic inhomogenous velocity field and Sawford and Stapountzis (1986) tested 1D and 2D meandering models in order to compare the concentration PDFs induced by a line source and a point source, respectively.

A significant step forward in the meandering models was made by Yee et al. (1994b), who included the contribution to the in-plume fluctuations due to the relative dispersion. To that aim, the authors proposed to parametrize $f_{\phi r}$ as a gamma distribution. This shape of PDF is not rigourosly justified, but is based on some heuristic and experimental considerations (see Sect. 4.3). We mention that other authors tested a lognormal distribution for $f_{\phi r}$ (Franzese 2003; Hilderman and Wilson 2007; Gailis et al. 2007).

The introduction of a more complex parametrization for $f_{\phi r}$ requires to model the relative intensity of concentration fluctuations, $i_{\phi r}= \sigma _{\phi r} / \langle \phi \rangle _{r}$. Most of the studies assumed $i_{\phi r}$ dependent on the *x*-coordinate only (i.e., 1D model) and constant across the (*y*, *z*) width of the plume (e.g., Yee et al. 1994b; Yee and Wilson 2000; Luhar et al. 2000; Cassiani and Giostra 2002b; Franzese 2003; Mortarini et al. 2009). This simplification provides reliable solutions close to the source, where the meandering is predominant. Conversely, it becomes unrealistic in the far field, where the relative dispersion is the main mechanism and $i_{\phi r}$ approximates the global fluctuation intensity $i_{\phi }$. In the far field, $i_{\phi r}$ is characterized by a U-shaped profile since the intermittency is lower in the plume centerline and larger on the edges (Gailis et al. 2007). The 1D model for $i_{\phi r}$ can be interpreted as a bulk or plume-averaged parameter at a particular downwind distance *x* from the source location that provides reasonable values of the scalar-field statistics (Reynolds 2000; Gailis et al. 2007; Mortarini et al. 2009). Three-dimensional models of $i_{\phi r}$ were tested by Gailis et al. (2007) and Marro et al. (2015) against some experimental datasets. The three-dimensional modelling was shown to be preferable when the meandering process is negligible with respect to the relative dispersion, namely when the the higher-order moments assume a bimodal shape (see Marro et al. 2015). Some aspects about the asymptotic behaviour of this upgraded fluctuating model deserve to be discussed. Neglecting the effect of relative dispersion ($i_{\phi r}\rightarrow 0$), the distribution for $f_{\phi r}$ is defined as in Eq. 32 (pure meandering model). Conversely, in the far field, the instantaneous plume centroid is basically located on plume centerline ($\sigma _{ym,zm} \rightarrow 0$) and $f_m$ tends to a Dirac delta function. Thus, the concentration PDF is only described by the relative dispersion process, i.e., $f_\phi \sim f_{\phi r}$, which is excellently reproduced by the gamma distribution.

Some more advanced applications of a fluctuating plume model included interference between two point sources (Yee et al. 2003), and chemical reactions (Ferrero et al. 2013).

Finally, Cassiani and Giostra (2002b) introduced a framework based on the linear transformation of the mean concentration field that extends the use of meandering plume models as a post-processor to any mean concentration field, that may be available from any modelling method or even experimental measurements. Cassiani and Giostra (2002b) applied this approach to dispersion in the convective boundary layer obtaining results equivalent to those of Luhar et al. (2000), but without any need to use stochastic Lagrangian model of particle trajectories and instead using the mean concentration obtained by a semi-analytical model (Cassiani and Giostra 2002a). Bisignano et al. (2017) applied this approach to the neutral wind-tunnel measurements of Nironi et al. (2015) obtaining satisfactory agreement for concentration moments up to the fourth order.

### Empirical Models for the Statistical Moments of Concentration

There is a class of closed relationships that, although are empirically obtained, have been shown to be a possible simpler and rapid alternative to demanding numerical simulations in some conditions. The main results originated from Chatwin and Sullivan (1990), who found that the central statistical moments of a passive scalar in self-similar turbulent flows (jets, wakes, and boundary layers), at large distance from the source, nicely follow

33 $$\begin{aligned} \langle \phi ^n \rangle = \beta ^n \frac{\phi _0^n}{\alpha }\left[ r(\alpha -r)^n+(-1)^n(\alpha -r)r^\alpha \right] , \end{aligned}$$

where $\phi _0=\langle \phi \rangle _{\text {max}}$ is the maximum mean value in a transversal/vertical section (in the centerline for a jet or a wake, and on the wall for a boundary-layer plume), and $r=\langle \phi \rangle /\phi _0$. From comparison with several experimental results, Chatwin and Sullivan (1990, (1993) proposed $1<\alpha <1.5$ and $0<\beta <1$. Equation 33 is particularly attractive because from the mean concentration field one may readily obtain higher-order moments in an expeditious way. Sawford and Sullivan (1995) explored the validity of Eq. 33 close to the source and found that the results are strongly dependent on the source configuration. They also suggested an additional parameter to Eq. 33 that can heuristically take into account the effect of molecular diffusion. Mole and Clarke (1995) additionally verified the empirical relationship by Chatwin and Sullivan (1990) with more experimental results, confirming the range of value for $\alpha$ and $\beta$. Mole and Clarke (1995) also pointed out that Eq. 33 leads to the simple relationship between the skewness $S_k$ and the kurtosis $K_u$

34 $$\begin{aligned} K_u=a S_k^2 + b, \end{aligned}$$

where *a* and *b* are $\sim$1, which depend on the experimental set-up (Schopflocher and Sullivan 2005). Equation 34 can be easily used to verify, for example, the properness of a PDF model (see Sect. 4.3).

Another class of semi-empirical models developed by Wilson et al. (1982a, (1982b, (1985) stemmed from Netterville (1979). Netterville (1979) proposed that the spatial distribution of the concentration variance was governed by a diffusion equation identical to that of the mean field, and that the effect of the ground could be accounted for through an image source. The main issue in such diffusion model is that production and dissipation of variance are neglected. Wilson et al. (1982b) argued that this deficiency can be compensated by defining a virtual location for the variance source to include the effects of production, and by an image sink to account for the increased dissipation near the surface. By doing so, and by setting some calibration parameters through experiments, Wilson et al. (1982b, (1982a), respectively, verified the model for the concentration variance with a ground-level and an elevated source. Wilson et al. (1985) later extended the analysis to the intermittency factor and the conditionally-averaged concentration fluctuation statistics.

### Analytical PDF Models

The complexity of turbulence has so far prevented from a theoretical solution for the PDF of concentration. Suitable numerical results may be obtained for a given experimental set-up through the demanding PDF methods (see Sect. 3.3). Yet, for practical purposes, a closed-form analytical function is needed to permit rapid calculations. Based on experimental and field data, several PDF models have been tested during the years for the concentration of a passive scalar released from a point source (see Table 2). Despite the effort, the question on which model better reproduces the concentration PDF remains open. In fact, the response usually depends on the experimental conditions.

One aspect of the concentration PDF that is somewhat controversial is related to the probability of $\phi =0$ (clean air). This might potentially be addressed through the intermittency factor $\gamma$, that is the probability of $\phi >0$. Yet, a correct definition of $\gamma$ remains elusive. In fact, although $\phi =0$ is a physically plausible value, it is in contrast with the description of scalar mixing according to the advection–diffusion equation, Eq. 2 (Chatwin and Sullivan 1989, 1993). Furthermore, the intermittency is not a precisely measurable quantity since its experimental value depends on a threshold that must be necessarily related to the instrument used for measuring the concentration (e.g., Fackrell and Robins 1982b) or, in case of odours, to the neurobiology of the olfactory system (Celani et al. 2014). Similarly, a threshold is also required in numerical simulations due to the presence of molecular and numerical diffusivities. Chatwin and Sullivan (1989, (1993) proposed an alternative definition of intermittency that is based on the representation of the concentration in a single realization neglecting the dissipative effects of molecular diffusivity, i.e., $\gamma =\langle \phi \rangle / \phi _s$ where $\phi _s$ is the unique value of the concentration at the marking source. Yet, the validity of this definition seems justified only in the very early phases of dispersion.

The mathematical representation of intermittency in the concentration PDF can also be the subject of a debate. Some authors (e.g., Lewellen and Sykes 1986a; Yee 1990; Yee et al. 1993c) use a Dirac delta function representation so that the concentration PDF is composed of two parts

35 $$\begin{aligned} f_\phi =\gamma f_{\phi >0}+(1-\gamma )\delta (\phi ), \end{aligned}$$

where $\delta$ is the Dirac delta. However, if a concentration threshold is considered, below which the value is either not measurable or unattainable, the integral of the concentration PDF below this threshold could be as well used to define the intermittency. Conveniently, the exact modelling of intermittency may be not relevant for several practical purposes that are linked to the high values of concentration and for which it is indifferent if the probability of low values of concentration lies exactly in $\phi =0$ or in a positive small interval. Our recommendation is that the explicit representation of a finite probability of $\phi =0$ shall be included in the PDF only if one is interested in that specific value or if this is necessary to correctly fit the calculated or observed concentration moments due to the choice of the model function representing the concentration PDF. The possible PDF models are the main subject of this section and are reviewed below.

The PDF models proposed in the literature (Table 2) are mostly two-parameter distributions, i.e., the first two statistical moments (mean and variance) provide the full concentration statistics. These models include: the clipped-normal, the gamma, the Weibull, the lognormal, and the beta. The only one-parameter distribution is the exponential, which is thus very appealing for practical purposes but it well resembles experimental data just close to the source of emission, i.e., in the meandering regime. The first comparisons between experimental data and PDF models suggested the clipped normal as best fitting distribution (see the first lines in Table 2). It was later shown that the clipped-normal does not universally reproduce the right skewness and the heavy upper tail of the concentration distribution (e.g., Yee et al. 1993c).

**Table 2:** Table extended from Efthimiou et al. (2016a)

Publication	Distributions	
Hanna (1984c)	Exponential	F
Lewellen and Sykes (1986b)	Clipped normal	F
Sawford (1987)	Clipped normal$^\mathrm{a}$, Exponential, Lognormal	F
Dinar et al. (1988)	Clipped normal$^\mathrm{a}$, Exponential	L
Yee (1990)	Clipped normal$^\mathrm{a}$, Exponential, Lognormal	F
Mylne and Mason (1991)	Clipped normal$^\mathrm{a}$, Exponential	F
Yee et al. (1993b)	Gamma$^\mathrm{a}$, Lognormal	F
Yee et al. (1993c)	Exponential, Gamma, Lognormal, Weibull$^\mathrm{a}$, Clipped normal, Conjugate beta, K-distribution	F
Lewis and Chatwin (1997)	Exponential and GPD	L
Yee and Wilson (2000)	Gamma	L
Luhar et al. (2000)	Gamma	L
Munro et al. (2001)	EVT	F
Lung et al. (2002)	Gamma$^\mathrm{a}$, Lognormal, Weibull$^\mathrm{a}$	F
Villermaux and Duplat (2003)	Gamma	L
Munro et al. (2003)	Beta and GDP	F
Yee and Biltoft (2004)	Clipped gamma$^\mathrm{a}$, Clipped normal	L
Gailis et al. (2007)	Gamma$^\mathrm{a}$, Lognormal	L
Yee et al. (2009)	Clipped gamma	L
Yee and Skvortsov (2011)	Gamma	L
Bartzis et al. (2015)	Beta	L
Nironi et al. (2015)	Gamma	L
Efthimiou et al. (2016a)	Lognormal, Gamma$^\mathrm{a}$	L
Oettl and Ferrero (2017)	Weibull, Gamma$^\mathrm{a}$, Lognormal	F

Models used to fit the concentration distributions from punctual sources in field (F) and laboratory (L) experiments

$^\mathrm{a}$Highlights the best fitting model(s)

More recent results have been converging on the choice of the gamma distribution as best PDF model for the concentration from a point sources at least over a certain range of downwind distances from the source (e.g., Yee and Skvortsov 2011; Marro et al. 2015; Ardeshiri et al. 2020),

36 $$\begin{aligned} f_{{\varGamma }}=\frac{\lambda ^{\lambda }\phi ^{\lambda -1}}{{\varGamma }[\lambda ]\langle \phi \rangle ^{\lambda }} \exp ({-\lambda \phi /\langle \phi \rangle }) \end{aligned}$$

where $\lambda =1/i_\phi ^2=\langle \phi \rangle ^2/\sigma _\phi ^2$ and ${\varGamma }[\cdot ]$ is the gamma special function (Abramowitz and Stegun 1965). Furthermore, experimental observations in confined turbulence (Villermaux and Duplat 2003; Duplat and Villermaux 2008) suggested the gamma distribution as a universal model in the context of passive scalars released from point sources.

As anticipated, the problem of theoretically deriving a solution for the concentration PDF remains unsolved. Yet, few notable works that undertook the issue may be mentioned. Csanady (1973, pp. 225–227) speculatively deduced a lognormal distribution assuming that the concentration is the product of a large number of independent dilution events induced by the turbulent eddies at a constant rate. A slightly different heuristical approach to obtain the lognormal in the meandering regime was proposed by Yee et al. (1993b, in the Appendix), and is based on the assumptions that a wide range of independent eddies carry the passive scalar. In the same Appendix, Yee et al. (1993b) also obtained a gamma distribution for the relative dispersion regime by assuming a Poisson distribution for the number of contaminated parcels contained in a finite volume of the well-mixed plume. Kowe and Chatwin (1985) obtained, under simplified assumptions, several solutions for the passive scalar PDF in the case of instantaneous cloud releases as a function of the axes of the rate of strain tensor. Yee and Chan (1997) derived a clipped-gamma distribution assuming ad hoc closures for what they called pseudo-diffusion and pseudo-dissipation terms. Chatwin (2002) presented empirical considerations to support the idea that, under simplifying assumptions, the PDF is inversely proportional to the mixing process by molecular diffusion. As Chatwin debated, this relation may serve in the modelling of the mixing term in the PDF transport equation. Villermaux and Duplat (2003) hypothesized that the stirring of stretched sheets leads to an aggregation process with a unique family of concentration distribution, i.e., the gamma distribution, stable by self-convolution.

The beta, as other clipped-distributions, has an upper limit for the concentration. This discloses the debate about whether the distribution model actually needs an upper limit. Theoretically, repeating a release event an infinite number of times, one could find a concentration equal to the source concentration at a certain point downwind the source (Wilson 1995). This could mean that no upper bound is needed for the values that the concentration can assume. However, the undiluted concentration downwind the source is so improbable that, for application purposes, the concentration may be considered bounded between a lower limit, which is usually zero or the background concentration, and an upper limit, physically given by the effect of molecular diffusion, which lowers the concentration below the value at the source (Munro et al. 2001). This upper bound can be defined through extreme value theory (EVT) (Munro et al. 2001, 2003) or empirical relationships (Efthimiou et al. 2017).

Extreme value theory may also be used to better reproduce the upper-tail of the concentration distribution. In fact, the PDF models are usually fitted to the bulk of the concentration data, so they do not necessarily perform well in the upper tail of the PDF, especially if high data values tend to be sparse. The EVT is a complex mathematical tool which can be used to reduce this issue (Munro et al. 2001), by assessing, from a given ordered sample of concentration peaks, the probability of events more extreme than the peaks previously observed. Through EVT, the Generalized Pareto Distribution GPD has been shown to fit the upper tail of the PDF in various field observations (Mole et al. 1995), laboratory experiments (Schopflocher 2001; Mole et al. 2008), and numerical approaches (Xie et al. 2004). Yet, the GPD does not have a structure complex enough to reproduced the full shape of concentration PDF (Schopflocher 2001), so several works have combined the GPD to other distribution function as the exponential (Lewis and Chatwin 1995), or the beta (Munro et al. 2003). We need to stress out that the uncertainty of the EVT methods remains very high (Munro et al. 2001), and that recently the gamma distribution has been shown to provide good estimates even for the upper tail of the concentration (Efthimiou et al. 2016a). Thus, the gamma distribution may be an easier-to-implement alternative.

Finally, a rather lower amount of research has been devoted to the definition of a proper model for the one-point one-time PDF for line sources of emission. This open challenge could be addressed starting from experimental (e.g. Stapountzis et al. 1986; Lavertu and Mydlarski 2005; Lepore and Mydlarski 2011) and numerical (e.g. Boppana et al. 2012) PDFs of passive scalar obtained in turbulent channels. Notably, Venaille and Sommeria (2008) have shown that in confined turbulence the gamma distribution (Eq. 36) does not correctly reproduce the PDF of the passive scalar emitted from a line source. They also experimentally observed that the validity of self-convolution models based on the aggregation of stretched sheets (Villermaux and Duplat 2003) is questionable even at low *Re* and strongly depends on the source of injection.

### Time-Series Models

At a fixed receptor, the concentration of a passive scalar dispersed in the atmosphere exhibits a strongly stochastic dynamics determined by the underlying turbulent velocity field (see Fig. 2). In several applications, as the assessment of hazards related to toxic substances or the level of annoyance induced by nuisance odours, a proper definition of the temporal characteristics of the concentration signals may be fundamental. Indeed, when we deal with these problems, even the full characterization of the concentration PDF is insufficient, and must be complemented with the knowledge of temporal quantities such as the mean frequency of exceeding a certain concentration threshold $\theta$ (upcrossing rate $N^+_\theta$). The upcrossing rates are key level-crossing statistics, as from them it is possible to readily calculate: the peak concentration in a given sampling time, the mean interval of time above a certain threshold (upcrossing time), and the mean waiting time (return period) (Yee et al. 1993a; Wilson 1995).

Several approaches have been adopted during the years to address the temporal characterization of the concentration signals. Among the first to tackle the problem, Högström (1972) used an experimentally-calibrated fluctuating plume model (see Sect. 4.1) to reproduce odour frequencies of field experiments in Sweden. The comparison between field data and theoretical results was imprecise, probably both for the complexity of the field experiment and the strong assumptions of the fluctuating model, for which Gaussian one-point one-time PDF and spatial distribution for the mean concentration were assumed. Yet, based on this notable attempt, similar models have been developed more recently (de Melo Lisboa et al. 2006; Dourado et al. 2014).

Another approach was proposed by Kristensen et al. (1989) and Yee et al. (1993a), who adopted Rice’s theory (Rice 1944) to relate the upcrossing rates to the joint PDF of concentration and its time derivative ($f_{\phi {\dot{\phi }}}$), or in alternative the PDF of the concentration time derivative conditioned to the $\theta$ threshold ($f_{{\dot{\phi }}\mid \theta }$). However, the definition of one of the two PDFs ($f_{\phi {\dot{\phi }}}$ or $f_{{\dot{\phi }}\mid \theta }$) may require several assumptions (Kristensen et al. 1989; Wilson 1995) and jeopardize the applicability of Rice’s theory.

Lately, the research has focused on stochastic models that reproduce the concentration time series (Du et al. 1999; Hilderman and Wilson 1999; Jones and Thomson 2006; Cassiani et al. 2009). In general, all these models require the PDF (or alternatively the mean and the intensity of concentration fluctuations) and a time scale (usually the Eulerian integral scale) to be set (both usually estimated from experiments or by means of empirical relations). More specifically, the stochastic model of Du et al. (1999) numerically reproduces a non-intermittent ($\phi >0$) concentration time series, and consequently allows for the evaluation of the upcrossing rates. Du et al. (1999) also showed that the assumption of a lognormal PDF, instead of a gamma PDF, provides a better estimate for the atmospheric upcrossing rates measured by Yee et al. (1993b). However, they also state that this could be a peculiarity of the dataset used for the model verification. Hilderman and Wilson (1999) further extended the model by Du et al. (1999) to intermittent signals. The stochastic model by Jones and Thomson (2006) uses a correlation-distortion technique that, starting from a PDF and an energy spectra, generates a Gaussian process with modified spectral characteristics, and eventually yield a non-Gaussian process with the desired spectral characteristics. The model by Iacono and Reynolds (2008) reproduces an observer who is randomly moving in an inhomogeneous plume, and is thus applied to the biological study of odour-mediated insect flights. Iacono and Reynolds (2008) also provided an analytical relationship for the Eulerian integral time scale, based on the field data by Yee et al. (1994a). Lastly, Cassiani et al. (2009) coupled a system of Eulerian stochastic equations for the velocity and concentration time series, to a Lagrangian PDF micromixing model. The Lagrangian model is used to obtain the concentration PDF and the concentration statistics conditioned on the velocity, which are required by the Eulerian model. Notably, consistent relationships are formulated between the Eulerian and Lagrangian dissipation time scales used in the models.

All the aforementioned stochastic models require a numerical approach to address the level crossing statistics. Analytical exceptions were provided by Yee (2000) and Bertagni et al. (2020), who, starting from the assumption of lognormal and gamma PDFs, respectively, derived closed form relationships for the upcrossing rates $N^+_\theta$ and other level-crossing statistics.

## Discussion

We have reviewed the advancement in the understanding of scalar concentration fluctuations from localized sources from the past 70 years, starting from the early experimental and modelling works in the mid 20th century to the most recent work.

A large number of experimental works have been performed both in the laboratory and in the field. Laboratory experiments have the main advantage of minimizing the uncertainties of the control parameters: the velocity field is statistically steady and the scalar flux at the source is perfectly known. In these conditions experimental errors are relatively easily evaluated. At the same time, these experiments are performed with Reynolds number that are lower than those characterizing real atmospheric flows, which may raise questions on how these are effectively representative of atmospheric dispersion processes. This issue does not exist in field measurements but, on the other side, there are higher experimental uncertainties in the results. This is mainly due to the fact that a proper steadiness of the flow is rarely achieved. Moreover, the complete characterization of the flow statistics typically available in laboratory experiments is seldom achieved in field experiments.

The literature cited in this review shows that there is a limited overlap between the configurations investigated in field and laboratory experiments. Most field experiments considered the case of extremely small sources (compared to boundary-layer thickness), mainly located at (or near) the ground with concentration measurements within the surface layer. Conversely, most laboratory experiments considered instead the case of an elevated source with a relatively ‘large’ size, and few experiments were actually devoted to the characterization of ground-level releases. A systematic investigation of the effects of source size is so far confined to laboratory experiments, since data for field campaigns are generally collected far away from the release location where source effects are lost. Effects of atmospheric stability (although confined to the surface layer) have been systematically investigated in field experiments, with the exception of very stable conditions. These effects have been instead investigated extremely rarely in laboratory experiments: very few laboratory measurements are available for unstable conditions and none in stable conditions. Finally, note that line-source experiments are so far confined to laboratory studies and are anyway relatively fewer compared to point-source studies.

All these features point out that there is a need in increase the overlap between laboratory and field measurements, which will certainly improve our confidence in the ability of laboratory experiments to correctly represent atmospheric dispersion and mixing. In this perspective, we think that it would be worth performing field experiments with elevated releases (of relatively large size), i.e., reproducing the configuration of laboratory experiments (Fackrell and Robins 1982a, b) that have been so far considered as a reference case for the validation of a wide range of modelling approaches. Developments of remote-sensing techniques and unmanned aerial vehicles equipped with micro-sensors are likely to fill this gap in the coming years. Beyond that, there is a clear need to improve the size of experimental datasets devoted to the characterization of concentration fluctuations in a variety of atmospheric stability conditions, both in the laboratory and in field experiments.

Despite their limitations, experiments performed so far have certainly elucidated the behaviour of concentration fluctuations, notably the second-order concentration moment. The evolution of higher-order moments and the overall PDF shape has still not been fully revealed. The gamma distribution has been so far proven to be the most suitable model for the concentration PDF from point sources over a wide range of downwind distances. Exceptions are the near-source region, where the concentration fluctuation intensity increases, and perhaps, the region where the concentration fluctuation intensity has nearly constant values. However, this is not the case for line sources for which the gamma PDF seems not to be a suitable model. Therefore, more research is necessary to fully elucidate the PDF shape and its representation in all phases of plume evolution.

On the modelling side, we have reviewed the available methods used to forecast concentration fluctuations at the very high Reynolds numbers typical of atmospheric flow. Among these, LES is certainly the most comprehensive approach, although LES is computationally very demanding and results are very sensitive to grid resolution and numerical methods used. These characteristics make the application for small localized atmospheric releases difficult and this is reflected in the limited literature thoroughly applying and evaluating LES capability for concentration fluctuations. Two-particle Lagrangian methods are also difficult to apply in realistic conditions and again this is reflected in the limited relevant literature. RANS, heuristic Lagrangian, and PDF transport equation methods appear to be the most useful modelling alternatives and have similar needs for parametrizations. They especially need a proper definition of the concentration variance dissipation/micromixing time scale. Current parametrizations of this time scale rely on inertial-range scaling arguments but also on empirical constants of uncertain generality. More research is needed to fully validate formulations and generality of these time-scale models. For instance, a rigorous method for incorporating geometrical dimensionality of the source in the formulations is lacking.

RANS and heuristic Lagrangian methods forecast only the first-order and second-order concentration moments while PDF transport equation method can potentially forecast moments of any order, although computational resources limit this theoretical approach. In many instances, the concentration PDF is needed and analytical formulations are often preferable to the numerical representation provided by a PDF transport equation method. The gamma PDF shape and some other models are defined by just first- and second-order concentration moments but they have limitations. More sophisticated and general PDF formulations may be therefore useful. However, formulations that are more general will need more parameters, and therefore knowledge of more concentration moments to set their values. We believe that a method suitable to obtaining concentration moments of order higher than the second and, at the same time, flexible enough to be useful in practical applications, is the PDF transport equation method. However, with the caveat of using a suitable mixing model. The IEM and IECM models do not seem adequate for this purpose while velocity-conditioned particle interaction models or stochastic mixing models may be a suitable alternative. A task for future research relates to validating the accuracy of these classes of mixing models in reproducing moments higher than the second and even the full PDF shape. Given the ever-increasing computational resources and recent improvement in mixing models, it is likely that the solution of the PDF transport equation will soon become a suitable (and perhaps preferable) modelling choice whenever a reduced response time is not the main criterion for model selection. On the other hand, we have also reviewed models that are more suitable when a rapid response is needed and limited computational resources are available. All of the approaches presented here are well adapted for dispersion over homogeneous terrain and in near-neutral stability. In the case of complex flow fields and variable stability conditions several of the methods are, however, not fully adapted. With the aim of giving some guidance to practitioners, we have summarized in Table 1 the actual state-of-the-art situation concerning the applicability of existing approaches. The fluctuating plume model remains a reliable alternative to more ‘expensive’ methods, but only in a downwind range very close to the source. Its application further away from the source should be adopted only when a minimal computational requirement is the main criterion for modelling selection. Similar considerations hold for semi-empirical methods.

If LES is not used and the time evolution of the turbulent signal is needed, as for example in threshold-crossing problems, models that compute one-point one-time statistics need to be supplemented by methods for providing the time evolution of the turbulent signal. The formulation of these models is relatively straightforward because all the necessary statistics and time scales are readily available after the (previous) run of the model for forecasting concentration moments. Note that the time scales used in a time-series model have to be consistent with the time scales used in models for concentration moments. Therefore, the time-series simulation seems a quite straightforward task. Nevertheless, to our knowledge, validation studies of time-series models are currently limited to neutral stability conditions and a flat homogeneous landscape. Further investigations are needed to evaluate their suitability in the case of more complex flow topologies and stability conditions.

To date, experiments, supplemented by a theoretical approach in homogeneous isotropic turbulence, have been the main tool to guide our understanding of concentration fluctuations in atmospheric turbulent flows. With the increase in computational resources, it is likely that DNS (despite still confined to relatively low Reynolds number) will play an increasing role in elucidating concentration fluctuations from localized atmospheric releases and a contribution may also come from LES if grid and numerical effects are properly characterized.

This review reveals that there are nowadays a variety of approaches in order to numerically estimate the statistics of concentration fluctuations downwind of a localized pollutant source. Despite the significant amount of work in this field in the last few decades, these approaches are not yet widely adopted by the practitioner for technical studies and real-world problems. In fact, even though dispersion models are worldwide used for the prediction of hourly-averaged pollutant concentration (mainly in the context of chronic risks assessments), models used to estimate concentration fluctuations are rarely adopted. There are two reasons for this: one is that these approaches are generally more complex than canonical dispersion models; the other, which is probably the most important, is that there are today very few legislative constraints that would make mandatory the adoption of such models. For example, in recent years, different attempts have been made to include prescriptions for odour thresholds that lead to the development of operational models for the estimate of odour concentration (Brancher et al. 2020). As evidenced in Brancher et al. (2017), however, these prescriptions vary significantly from one country to another, and even between different regions of the same country. Furthermore, most of the time, the adoption of such prescriptions is still considered as optional according to existing laws. Therefore, the spread of knowledge required to apply approaches for the estimate of concentration fluctuations has yet to occur in the technical community. It is our hope that this review will contribute in this regard.

We have not covered in detail the related problem of concentration fluctuations for extensive area sources, e.g., forests or urban areas. Whether these need to be treated as localized or extensive area sources is perhaps a matter of scales, but most importantly, in these cases the role of concentration fluctuations is mainly relevant in conjunction with atmospheric chemical processes, an aspect that has not been addressed here. Nonetheless, at least two of the techniques reviewed here can be naturally extended to handle the influence of fluctuations on chemical reactivity: the PDF methods reviewed in Sect. 3.3 and the LES method reviewed in Sect. 3.1, the latter with the caveat of high resolution. An early example of the application of PDF methods to extended planar sources is Cassiani et al. (2005c) and an early example of application of LES is Patton et al. (2000). Between the two, the PDF method is certainly less computationally demanding (Pope 2000).

Finally, we would like to remark that the review spans a wide number of experimental works and covers modelling approaches of quite different natures. While preparing it we found several works of which we were previously unaware, and probably relevant papers have been missed. We apologize accordingly.

## Appendix

The Lagrangian particle approach to turbulent dispersion in the atmosphere dates back to the seminal work of Taylor (1922) and the historical evolution of this approach is very well captured in the review of Thomson and Wilson (2013). Lagrangian single-marked-particle models (e.g., Thomson 1987; Wilson and Sawford 1996) are routinely used for calculating the motion of an independent marked particle which suffices to calculate the mean concentration of a conserved scalar. Recalling that the concentration carried by a marked Lagrangian particle is conserved (see e.g., Monin and Yaglom 1971), i.e., $d \phi / d t=0$, we may write (Thomson 1987) for the mean concentration of a conserved scalar

37 $$\begin{aligned} \left\langle \phi (\varvec{x},t) \right\rangle = \int _{t_0\le t} \int _{V} p(\varvec{x},t;\varvec{x}_0,t_0)S(\varvec{x}_0,t_0)d\varvec{x}_0 dt_0, \end{aligned}$$

where $S(\varvec{x},t)$ is a scalar source function, the space integral extends over the whole volume, and $p(\varvec{x},t;\varvec{x}_0,t_0)$ is the probability that a marked particle that is in $\varvec{x}_0$ at the initial time $t_0$ will be in $\varvec{x}$ at the final time *t*. Lagrangian single-marked-particle models are used to compute this transition density. In the simpler form of random displacement models, also called zero-th order models (e.g., Thomson and Wilson 2013), a stochastic equation can be written for the position of a wandering particle ($\varvec{x}^*$),

38 $$\begin{aligned} d x_i^* = \left\langle u_i \right\rangle dt + \frac{\partial K}{\partial x_i} dt + \sqrt{2 K} dW_i, \end{aligned}$$

where $*$ denotes a particle quantity, *K* is the turbulent diffusivity, and $dW_i$ is an uncorrelated Wiener process with zero mean and variance *dt* (see e.g., Pope 2000). The solution of this equation for an ensemble of independent particles passing through the source can be used to calculate the transition density and, according to Eq. 37, the mean concentration. We note that, by neglecting molecular diffusivity and closing the turbulent fluxes by eddy diffusivity, i.e., $\left\langle u'_i \phi ' \right\rangle = -K \partial \left\langle \phi \right\rangle /\partial x_i$, Eq. 38 corresponds to a Lagrangian solution of Eq. 3, see e.g. Monin and Yaglom (1971, Sect. 10.3).

In recent years, most commonly, numerical Lagrangian dispersion models have been formulated using stochastic equations for the particle velocity and not just the particle position. This approach has developed in the atmospheric dispersion community to overcame the limitations of the gradient diffusion approximation for both the dispersion near the sources and in unstable stratification (e.g., Thomson 1987; Thomson and Wilson 2013). Including the particle velocity, the Lagrangian equations for the dispersing particle have the general form

39 $$\begin{aligned} d x_i^* = u_i^* dt, \end{aligned}$$

40 $$\begin{aligned} d u_i^* = a_i(\varvec{x}^*,\varvec{u}^*,t) dt + b_{ij}(\varvec{x}^*,t) dW_j. \end{aligned}$$

This system of equations for particle velocity and position correspond to a Fokker–Plank (transport) equation for the evolution of the single-time single-point velocity PDF (e.g., Thomson 1987). By assuming an analytical shape for the PDF of the velocity, $f_u$, the drift coefficient $(a_i)$ is usually defined through the well-mixed approach proposed by Thomson (1987). It is worth noting that the proper parametrization of $a_i$ is necessary to ensure that, at a fixed point, the statistics computed in the Lagrangian framework are equal to the Eulerian statistics. In the atmospheric boundary layer the analytical shape of the velocity PDF can be assumed Gaussian in neutral and stable conditions, and a skewed sum of two Gaussian in convective conditions (e.g., Thomson 1987; Stull 1988; Rodean 1996; Luhar et al. 1996; Rotach et al. 1996; Cassiani et al. 2005c, 2015; Thomson and Wilson 2013). For a neutral boundary layer with negligible cross-correlations $\langle u'_i u'_j \rangle$, a possible formulation of the drift term $a_i$ is

41 $$\begin{aligned} a_i = -\frac{{u'_i}^*}{T_{Li}}+\frac{1}{2} \frac{\partial \sigma _{ui}^2}{\partial x_i} + \frac{{u'_i}^*}{2\sigma _{ui}^2} \left( u_j^*\frac{\partial \sigma _{ui}^2}{\partial x_j}\right) , \end{aligned}$$

where ${u'_i}^*$ are the Lagrangian velocity fluctuations and $T_{Li}$ are the Lagrangian integral time scales and no summation is implied on repeated *i* indexes. Formulations of $a_i$ for the convective boundary layer can be found in Luhar et al. (1996) and in Cassiani et al. (2015). The diffusion coefficient $b_{ij}$ is determined by consistency with Kolmogorov similarity theory for the Lagrangian structured function (Thomson 1987) as originally suggested by Obukhov (1959) (see also Monin and Yaglom 1971) and it is generally defined as

42 $$\begin{aligned} b_{ij}=\delta _{ij}\sqrt{C_0\varepsilon }, \end{aligned}$$

where $\delta _{ij}$ is the Kronecker delta and $C_0$ is the Kolmogorov constant.

## List of Symbols

$\alpha$: Empirical constant in Eq. 33
$\alpha _{1,2}$: Empirical constants in Eqs. 8 and 10
$\beta$: Empirical constant in Eq. 33
$\delta$: Dirac delta function
$$\eta $$: Kolmogorov’s microscale
$\nu$: Kinematic viscosity of air
$$\gamma $$: Intermittency factor
$${\Delta } t$$: Sampling time
$\nu$: Kinematic viscosity of air
$\phi$: Passive scalar concentration
${\varPhi }_c$: Conditional averaged scalar concentration
$\phi _s$: Concentration at the source
$\sigma$: Total plume spread
$\sigma ^2_{\phi ,m}$: Variance of $\phi$ induced by meandering motion
$\sigma ^2_{\phi ,r}$: Variance of $\phi$ induced by relative dispersion
$$\sigma ^2_{\phi }$$: Variance of $$\phi $$
$$\sigma ^2_{u,v,w}$$: Variance of *u*, *v*, *w*
$\sigma _m$: Plume spread induced by meandering
$\sigma _r$: Plume spread induced by relative dispersion
$\sigma _{\varvec{u}r}$: Root-mean square of the relative velocity $\varvec{u}_r$
$\tau$: Integral time scale of the concentration
$\tau _m$: Mixing time scale
$$\theta $$: Concentration threshold
$\varepsilon$: Dissipation rate of turbulent kinetic energy
$\varepsilon _{\phi }$: Dissipation rate of the scalar variance
$$\varvec{u}$$: Velocity vector $$\varvec{u}=(u,v,w)=(u_1,u_2,u_3)$$
$\varvec{u}_r$: Difference between the turbulent velocity and the velocity of the instantaneous centre of mass
$$\varvec{x}$$: Coordinate vector $$\varvec{x}=(x,y,z)=(x_1,x_2,x_3)$$
$\lambda _D$: Decay rate for reacting scalar
${\varOmega }$: Generic mixing model Eq. 20
*a*: Order 1 parameter in Eq. 34
$a_{i}$: Drift coefficient in Eq. 20
*b*: Order 1 parameter in Eq. 34
$B_{ij}$: Diffusion coefficient in Eq. 20
$b_{ij}$: Diffusion coefficient in Eq. 40
$$C_0$$: Komogorov constant
$C_r$: Richardson–Obukhov constant
*D*: Molecular diffusivity
$$d_0$$: Source size
$d_r^2$: Parametrized inertial range relative dispersion
*E*: Turbulent kinetic energy
$f_\phi$: One-time one-point concentration PDF
$f_m$: PDF of meandering plume centre of mass position in the crosswind plane
$f_{\phi r}$: One-time one-point concentration PDF in relative coordinates
$f_{\phi u}$: Joint velocity–scalar concentration PDF
$h_b$: Boundary-layer height
$$h_s$$: Source height
$i_{\phi m}$: Intensity of concentration fluctuations due to meandering
$i_{\phi r}$: Intensity of concentration fluctuations due to relative dispersion
$i_{\phi }$: Intensity of concentration fluctuations
*K*: Turbulent diffusivity of mean concentration field
$K_{\phi ^{'2}}$: Turbulent diffusivity of the variance of concentration field
*L*: Turbulent length scale
$L_\phi$: Puff or plume size
$m_i$: Mass of a particle *i*
$N^+_\theta$: Mean frequency of upcrossing the threshold $\theta$
$N_j$: Number of particles within a cell *j*
$p(\varvec{x},t;\varvec{x}_0,t_0$): Probability for a particle to be in $\varvec{x}$ at *t* starting from $\varvec{x}_0$ at $t_0$
$p^{A,B}$: Probability for two particles *A* and *B* to be in $\varvec{x}^A,\varvec{x}^B$ at $t^A,t^B$ starting from $\varvec{x}_0^A,\varvec{x}_0^B$ at $t_0^A$, $t_0^B$
*Re*: Reynolds number
*S*: Source term
$$S_t$$: Turbulent spectrum
*Sc*: Schmidt number
*T*: Turbulent time scale
$t_0$: Characteristic time of the source size
$T_\phi$: Scalar dissipation time scale
$T_a$: Mean advective travel time
$T_L$: Lagrangian integral time scale
$VC_j$: Volume of a grid cell *j*
$vp_i$: Volume of a particle *i*
*x*: Longitudinal coordinate
$$x_i$$: *i*-Coordinate
*y*: Transversal coordinate
*z*: Vertical coordinate
$${\bar{\cdot }}$$: Time average
$\cdot '$: Fluctuation with respect to the mean value
$\langle \cdot \vert \cdot \rangle$: Ensemble conditional average
$$\langle \cdot \rangle $$: Ensemble average
$${\tilde{\cdot }}$$: Volumetric average
$${\cdot }^*$$: Lagrangian quantity
DNS: Direct numerical simulation
EVT: Extreme value theory
GPD: Generalized Pareto distribution
IECM: Interaction by exchange with the conditional mean
IEM: Interaction by exchange with the mean
LES: Large-eddy simulation
LIF: Laser induced fluorescence
PDF: Probability density function
PIV: Particle image velocimetry
SGS: Subgrid scale
